# *In silico* identification of rescue sites by double force scanning

**DOI:** 10.1093/bioinformatics/btx515

**Published:** 2017-08-14

**Authors:** Matteo Tiberti, Alessandro Pandini, Franca Fraternali, Arianna Fornili

**Affiliations:** 1 School of Biological and Chemical Sciences, Queen Mary University of London, London, UK; 2 Department of Computer Science, College of Engineering, Design and Physical Sciences and Synthetic Biology Theme, Institute of Environment, Health and Societies, Brunel University London, Uxbridge, London, UK; 3 Randall Division of Cell and Molecular Biophysics, King‘s College London, London, UK; 4 The Francis Crick Institute, London, UK; 5 The Thomas Young Centre for Theory and Simulation of Materials, London, UK

## Abstract

**Motivation:**

A deleterious amino acid change in a protein can be compensated by a second-site rescue mutation. These compensatory mechanisms can be mimicked by drugs. In particular, the location of rescue mutations can be used to identify protein regions that can be targeted by small molecules to reactivate a damaged mutant.

**Results:**

We present the first general computational method to detect rescue sites. By mimicking the effect of mutations through the application of forces, the double force scanning (DFS) method identifies the second-site residues that make the protein structure most resilient to the effect of pathogenic mutations. We tested DFS predictions against two datasets containing experimentally validated and putative evolutionary-related rescue sites. A remarkably good agreement was found between predictions and experimental data. Indeed, almost half of the rescue sites in p53 was correctly predicted by DFS, with 65% of remaining sites in contact with DFS predictions. Similar results were found for other proteins in the evolutionary dataset.

**Availability and implementation:**

The DFS code is available under GPL at https://fornililab.github.io/dfs/

**Supplementary information:**

Supplementary data are available at *Bioinformatics* online.

## 1 Introduction

A single amino acid change can significantly disrupt the function of a protein by affecting its structure, dynamics and stability and the interaction with its partners. Indeed, a wide range of inherited and somatic diseases has been associated with protein missense mutations (Gao *et al.*, 2015; Lu *et al.*, 2015; Studer *et al.*, 2013). Interestingly, it has been shown that inactive mutants can be partially or totally reactivated by additional mutations occurring at different positions of the same protein (intragenic rescue or suppressor mutations) (Baroni *et al.*, 2004; Kondrashov *et al.*, 2002; Poon *et al.*, 2005). Early studies estimated that ∼80% of deleterious mutations in an organism can be compensated by at least another mutation and ∼80% of these suppressor mutations occur in the same gene as the original mutation (Poon *et al.*, 2005). A prototypical example of protein that can be reactivated is the tumour suppressor p53: several mutations have been found to restore p53 function after disruption by single pathogenic mutations in the core DNA binding domain (Joerger *et al.*, 2005).

A remarkable aspect of rescue mutations is that they can be exploited for drug discovery (Baroni *et al.*, 2004; Chen *et al.*, 2010). Indeed, the spatial location of rescue mutations in the p53 structure has been used as a guide to design new anti-cancer drugs. For example, a region previously identified as rich in rescue mutations was recently targeted in a computer-aided drug discovery study (Wassman *et al.*, 2013). The predicted compound was shown to effectively reactivate a severely compromised p53 mutant in human cancer cells.

In principle, rescue mutations could be detected by systematically testing with specific functional assays all the possible combinations of double or multiple mutations in a protein, but this can easily result in a very large number of mutants to be assessed (Baronio *et al.*, 2010). The discovery of new rescue mutations could be accelerated by *in silico* tools able to perform complete screenings and to identify the best candidates for further experimental validation. For drug design, it would be particularly important to identify rescue sites that act through intra-molecular mechanisms, i.e. that can rescue the protein function by recovering intrinsic properties of the protein and not by directly interacting with partners. Indeed, these sites are more likely to be successfully mimicked by a small molecule without the need to interfere with protein–protein interactions.

Despite their importance, no general method based on physical principles is currently available for the computational prediction of intra-molecular rescue sites. Predictors based on machine learning methods are available, but only for specific cases such as p53, where a large amount of experimental information on rescue mutants could be used to train the model (Danziger *et al.*, 2009; Huang *et al.*, 2011; Ramani and Jacob, 2013).

The comparison of human and non-human sequences of evolutionarily related proteins has provided some insight into rescue mechanisms observed during evolution (evolutionary compensatory mechanisms) (DePristo *et al.*, 2005; Poon *et al.*, 2005). In particular, compensated pathogenic mutations have been found, where the mutated amino acid is part of the wild type sequence of non-human homologues (Barešić *et al.*, 2010; Ferrer-Costa *et al.*, 2007; Kondrashov *et al.*, 2002). Sequence and structural analyses of these mutations have provided information on their frequency, type of amino acid change and location in the 3D structure. However, in this type of studies (i) it is difficult to identify with certainty the compensatory mutations associated to a specific compensated pathogenic mutation, because of the large number of divergent positions usually found in the alignments of human and non-human sequences (Kondrashov *et al.*, 2002); (ii) it is not possible to distinguish between compensatory mechanisms mediated by inter-molecular and intra-molecular effects (Ferrer-Costa *et al.*, 2007); (iii) not all the possible cases of functional rescue are analysed, but only those fixed during evolution and (iv) compensatory effects are often assumed to be short-ranged or local (Barešić *et al.*, 2010; Ferrer-Costa *et al.*, 2007). Although this last assumption might be true in many cases, global suppressor mutations, i.e. mutations that can rescue multiple deleterious mutations, have been observed in several proteins (DePristo *et al.*, 2005). Moreover, allosteric rescue mutations have been observed in different cases (Dokholyan, 2016; Liu and Nussinov, 2008).

In this work, we introduce a general method to identify potential rescue sites that uses intra-molecular mechanisms mediated by backbone dynamics. The method is based on the simultaneous perturbation of candidate pathogenic-site/rescue-site pairs through the application of external forces and the measurement of the resulting structural variation. All the possible pairs in the protein are scanned and a rescue effect is detected when the combined perturbation of the two sites affects the protein structure less than the single perturbation of the pathogenic site. This approach, which in the following will be referred to as double force scanning (DFS), is unique in that (i) it is based on first principles, with no parameters introduced to reproduce specific rescue effects, (ii) only the native structure of the protein is needed as input and (iii) no assumption is made on the distance between rescue and rescued sites.

DFS predictions were compared with reference rescue sites, derived both from a dataset of experimental rescue mutants of p53 and from a dataset of evolutionary compensatory mutations in 10 different proteins. Remarkably, about half of the rescue sites in p53, including the global suppressor region, were predicted by DFS, with 65% of remaining rescue sites in close contact with DFS predictions. Similar results were found for other proteins in the evolutionary dataset.

## 2 Materials and methods

### 2.1 The DFS method

The DFS method is designed to detect rescue sites in a protein i.e. residues that upon mutation can counteract the effects of deleterious mutations at different sites. The method focuses on rescue sites that use intra-molecular mechanisms mediated by backbone dynamics and it is based on the assumption that the structural perturbation induced by a mutation can be mimicked by the application of a force (Atilgan *et al.*, 2012; Echave and Fernández, 2010). Perturbations induced by external forces have been previously shown to be compatible with the structural changes associated with evolutionary structural divergence (Echave, 2008), random mutations (Echave and Fernández, 2010) and ligand binding (Atilgan and Atilgan, 2009).

**Fig. 1.**
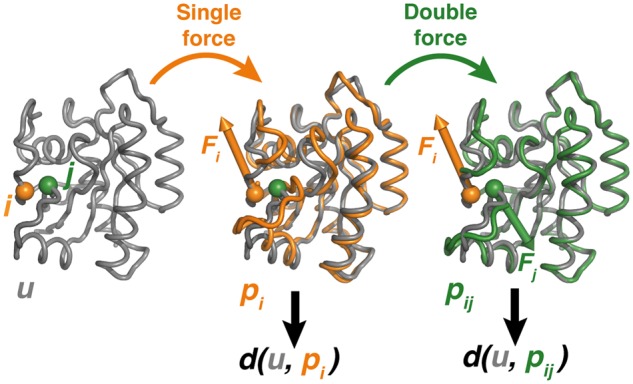
Schematic representation of the DFS approach. The protein unperturbed structure (*u*) is represented as grey tube, together with the structures after application of a single force **F_i_** at *i* (*p_i_* structure, orange) and of two forces **F_i_** and **F_j_** at *i* and *j*, respectively (*p_ij_* structure, green). The deviation of structure *x* from the unperturbed structure is measured by a distance function *d*(*u, x*) (Color version of this figure is available at *Bioinformatics* online.)

In the DFS method, we simultaneously apply forces mimicking the pathogenic mutation at a first site (residue *i*) and the candidate rescue mutation at a second site (residue *j*) and compare the resulting structural modifications to the ones produced by a single force at the first site (Fig. 1). The compensatory effect is quantified by the rescuability index $\rho_{ij}=\left(d\left(u,p_{i}\right)-d(u,p_{ij})\right)/d(u,p_{i})$, where *u* represents the native unperturbed structure, *p_i_* and *p_ij_* the structures upon application of the single and double forces, respectively, and *d(x, y)* is a distance function measuring the difference between structures *x* and *y*. A value of *ρ* between 0 and 1 indicates that the structural perturbation induced by the two forces is smaller than the one produced by the single force, thus highlighting the presence of a compensatory effect.

The effect of forces on the structure was simulated using the linear response theory (LRT) (Atilgan *et al.*, 2012; Echave and Fernández, 2010; Ikeguchi *et al.*, 2005) applied to the anisotropic network model (ANM) (Eyal *et al.*, 2006), where protein residues are described as single nodes (C^α^ atoms), connected by springs if their distance is smaller than a cutoff value *r_c_*. Within this framework, the effect of a perturbation is determined analytically using ${\Delta R\;=\;H}^{-1}F$, with Δ**R** indicating the vector of C^α^ atom displacements $\left\{\cdots ,{\Delta r}_{i}^{x},{\Delta r}_{i}^{y},{\Delta r}_{i}^{z},\ldots \}\right\}$, **F** the vector of external forces $\left\{\cdots ,F_{i}^{x},F_{i}^{y},F_{i}^{z},\ldots \}\right\}$ and **H** the ANM hessian.

Forces with different orientations can produce different structural perturbations (Supplementary Fig. S1A), making the value of *ρ_ij_* dependent on the relative orientation of the forces applied at the two sites. To take this into account, the following procedure was followed (see Supplementary Methods and Supplementary Fig. S2 for a schematic representation):

- for a given first-site force vector **F_i_**, multiple orientations of the second-site force **F_j_** are sampled and the rescuability index *ρ_ij_*is calculated for each of them. The maximum value of *ρ_ij_* (*ρ_i_*^MAX^) is recorded.
- the *ρ_i_*^MAX^ values are recorded for different orientations of the first-site force **F_i_** and an overall rescuability score *S_ij_* is calculated as their average.

In other words, the final rescuability score S_*ij*_ was calculated by maximizing *ρ_ij_* over the set of second-site force orientations (**F_j_)** and by averaging the resulting values over the set of first-site force orientations (**F_i_**). To avoid any bias, the set of force vectors on each site was generated so that the distribution of their orientations was as uniform as possible using the spherical Fibonacci lattice (Keinert *et al.*, 2015) (Supplementary Information). The relative magnitude of the forces applied on the two sites was determined using two different schemes. In the Fixed Force (FF) scheme the same magnitude was used for both **F_i_** and **F_j_**, while in the Fixed RMSD (FR) scheme, force magnitudes were rescaled so that they produced structural perturbations of the same magnitude when applied singularly, i.e. *d(u, p_i_)* = *d(u, p_j_).* Scores for all pairwise comparisons were collected in two rescuability matrices **S^FF^** and **S^FR^**, which were then combined in a unique score called the compensatory power *P*. First the number of residues rescued by a given second-site residue *j* (i.e. for which *S_ij_* > 0) was calculated from each matrix (**S^FF^** or **S^FR^**) and divided by the number of contacts of *j*. All the residues *i* in the protein were considered in this calculation. The resulting values were then rescaled from 0 to 1 and their average was used as the overall compensatory power *P*. Residues with higher *P* values have a higher potential of rescuing first-site residues according to **S^FF^** and/or **S^FR^** scores. The compensatory power *P* was used to predict experimental rescue sites in p53 and evolutionary rescue sites in a subset of the compensated pathogenic deviation (CPD) database as described below.

The DFS method was implemented in Python using the ProDy (Bakan *et al.*, 2014) package. Further details of the method are provided as Supplementary Material.

### 2.2 Database of rescue mutations in p53 and CPD80

A database of pathogenic and rescue mutations for p53 was derived from the available literature (Supplementary Table S1 and Supplementary Material). A dataset containing pathogenic sites (PS) and rescue sites (RS_exp_) was then generated from the position of the pathogenic and rescue mutations in the p53 sequence (Supplementary Table S2).

The CPD80 dataset (Supplementary Table S3) was extracted from the CPD database (Barešić *et al.*, 2010), where a pathogenic mutation in a human protein is annotated as compensated if the mutated amino acid occurs in the wild type sequences of non-human, functionally equivalent proteins. The non-pathogenicity of the mutated amino acid in the non-human protein is assumed to be due to the other differences between the human and non-human sequences. In this work we considered only proteins with close non-human homologues (sequence identity > 80%), to reduce the possibility that the differences in the sequences were not related to compensatory mechanisms. A final dataset of 10 proteins was considered (CPD80, Supplementary Table S3), where putative compensatory sites (evolutionary-related rescue sites or RS_evol_) were detected by identifying the positions with different amino acids in pairwise alignments of the human and non-human homologues (Supplementary Table S4 and Supplementary Material).

### 2.3 Performance analysis

DFS was run on each protein with the setup described in the Supplementary Material. Since the focus of the method is the prediction of intra-molecular rescue sites, a DNA-free structure was considered for p53 (PDB ID: 1TRS, chain A). Similarly, CPD80 structures were selected so that they contain the smallest biological unit of the protein and they are as representative as possible of an unbound state (Supplementary Material).

All the residues in each protein were ranked by DFS compensatory power *P* and residues with *P* greater than a threshold were classified as DFS rescue sites. The performance of the method at a given threshold was expressed in terms of sensitivity, specificity and accuracy. An optimal threshold (0.491, corresponding to the top 28% residues) was calculated by minimizing the quantity D = (1 − sensitivity)^2^ + (1 − specificity)^2^ for p53 (Hajian-Tilaki, 2013). This percentile value (top 28%) was selected as the reference threshold for all the DFS calculations performed in this work, since the p53 dataset is based on direct experimental evidence of functional reactivation. A discussion of the dependence of the threshold from the size of the dataset is included in the Supporting Material.

The enrichment of DFS predictions in experimental rescue sites with respect to to the whole set of residues was calculated as P_DFS_/P_random_, where P_DFS_ = TP/(TP + FP) is the fraction of DFS predictions that are classified as experimental rescue sites and P_random_ = (TP + FN)/nres is the fraction of residues in the protein that are classified as experimental rescue sites (where TP is the number of true positives, FP the number of false positives, FN the number of false negatives and nres the total number of residues).

All performance parameters were calculated using exact matches between predicted and reference data unless otherwise stated. All analyses were performed in R (https://www.R-project.org) using the package ROCR (Sing *et al.*, 2005) and Bio3D (Skjærven *et al.*, 2014).

## 3 Results

In the following sections we will describe the performance of DFS in predicting rescue sites for p53 and CPD80 proteins. In particular, we will analyse the ability of the method to identify the regions on the protein surface with a high density of rescue sites, since this is particularly relevant for the detection of binding pockets for compensatory drugs.

### 3.1 Prediction of rescue sites in p53

**Fig. 2.**
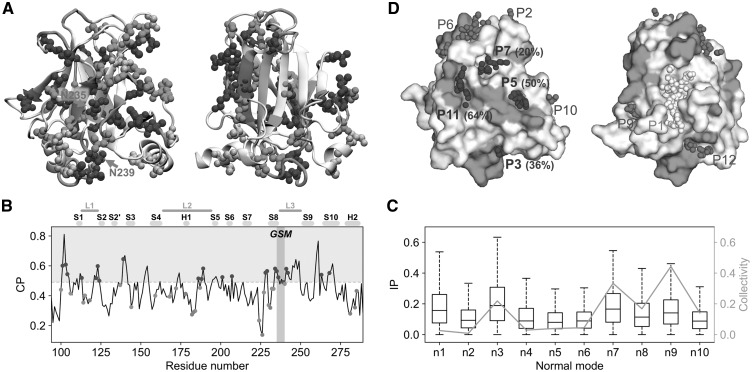
Detection of p53 rescue sites with DFS. **(A)** Comparison of experimental rescue sites and DFS predictions mapped onto the p53 structure. Experimental rescue sites (RS_exp_) are reported as spheres, coloured in blue (predicted by DFS), cyan (within 4 Å from DFS rescue sites) and grey (not predicted). The position of all DFS rescue sites is indicated with green cartoon. Two different views of the structure are shown, representing the sides that are rich (left) and depleted (right) in rescue sites. Two residues from the GSM are labelled in magenta. **(B)** Plot of the compensatory power *P*. The threshold *P_cut_* used for the definition of DFS rescue sites is represented with a dashed grey line. Experimental rescue sites RS_exp_ are indicated with dots coloured as in panel A. Secondary structure elements are indicated with gold (strands) and grey (helices) blocks, while loops L1–L3 are indicated with brown lines. The position of the GSM residues is shaded in magenta. **(C)** Distributions of inner products calculated between the compensatory motions and each of the first 10 normal modes represented as boxplots. The collectivity index of each normal mode is reported in orange. **(D)** Surface representation of p53, with DFS rescue sites coloured in green. Binding pockets detected with fpocket are reported showing the centres of the probe spheres used for their detection (α-spheres), coloured in blue (%RS_DFS_ and %RS_exp_ ≥ 20), green (%RS_DFS_ ≥ 20), grey (%RS_exp_ ≥ 20) and white (%RS_DFS_ and %RS_exp_ < 20). The content in DFS rescue sites (%RS_DFS_) is shown in parentheses for blue pockets (Color version of this figure is available at *Bioinformatics* online.)

To date, several p53 rescue mutants have been found and directly tested for functional reactivation (RS_exp_ dataset, Supplementary Table S1) (Baroni *et al.*, 2004; Baronio *et al.*, 2010; Brachmann *et al.*, 1998; Danziger *et al.*, 2009; Inga and Resnick, 2001; Joerger *et al.*, 2005; Merabet *et al.*, 2010; Odell *et al.*, 2013; Otsuka *et al.*, 2007; Wieczorek *et al.*, 1996). Mapping their positions onto the structure of p53 (spheres in Fig. 2A) highlights a non-uniform spatial distribution, with a side of the β-sandwich (left panel) richer in RS_exp_ residues than the other (right panel). The RS_exp_-rich side includes residues from the S8 strand and nearby L1, L2 and L3 loops. In particular, it contains the global suppressor motif (GSM) that includes residues N235 (S8) and N239, S240 (L3), which were shown to rescue a large number of pathogenic mutants (Baroni *et al.*, 2004).

The rescue sites predicted by DFS were compared with the experimental sites by mapping them onto the p53 structure (green cartoon in Fig. 2A) and sequence (green shading in Fig. 2B). The RS_exp_ sites (spheres in Fig. 2A and dots in Fig. 2B) were coloured in blue when they were predicted by DFS as rescue sites and in cyan when they were in contact with DFS predictions. Remarkably, predicted rescue sites were found to follow the same spatial distribution as the experimental ones, clustering in the RS_exp_-rich regions and including the GSM (purple in Fig. 2B). The performance analysis (Table 1) showed that about half (47%) of the RS_exp_ residues were predicted as rescue sites by DFS. Of the remaining 26 RS_exp_ positions, 17 of them were in contact (within 4 Å) with DFS-predicted sites, so that 82% of the RS_exp_ sites were either predicted by DFS or in contact with DFS predictions (Supplementary Tables S5 and S6). A high specificity was also found, with 78% of the non-rescue residues correctly classified as such. Overall, the probability of finding a true rescue residue among DFS predictions was 1.67 times larger than that of a random guess. It is also to be noted that, while the compensatory power profile discussed earlier was obtained on a DNA-free conformation of p53, it showed a good stability for small changes in the structure (Supplementary Fig. S3 and Supplementary Material).

**Table 1.** DFS performance for p53 and CPD80 proteins

Protein	Sensitivity^a^	Specificity^a^	Accuracy^a^
p53	0.469	0.782	0.704
SOD	0.435	0.758	0.693
AT-III	0.294	0.721	0.670
AAT	0.214	0.718	0.699
KRAS	0.571	0.730	0.723
TTR	0.362	0.744	0.648
ALB	0.288	0.722	0.645
TBG	0.247	0.711	0.622
AAC1	0.265	0.715	0.641
IL13	0.400	0.722	0.708
TPMT	0.213	0.696	0.568
<CPD80>^b^	0.329	0.724	0.662
<p53 + CPD80>^c^	0.342	0.729	0.665

a Values obtained setting the cutoff on the compensatory power *P* to the top 28th percentile (0.491).

b Average over CPD80 proteins.

c Average over p53 and CPD80 proteins.

Since DFS was developed to detect specific compensatory mechanisms, it is not surprising that not all the known p53 rescue sites are recovered. Indeed, some of the RS_exp_ residues that were not classified as rescue sites by DFS are known to use compensatory mechanisms that are less likely to rely on backbone motions. Examples of such residues are H168, T284 and H178 (Supplementary Table S6). Indeed, the H168R mutation is thought to rescue R249S by side chain mimicking, i.e. by simply replacing the R side chain lost when the pathogenic mutation occurs (Joerger *et al.*, 2005), while rescue mutations at T284 and H178 are directly involved in inter-molecular interactions with DNA (T284R) (Wieczorek *et al.*, 1996) and other p53 monomers (H178Y) (Otsuka *et al.*, 2007).

In order to get a deeper insight into the compensatory mechanisms detected by DFS, we analysed the double-force displacements producing the largest values of the rescuability indices ρ for each residue pair (‘compensatory motions’) in terms of the first 10 normal modes of the unperturbed protein structure (‘essential space’). The degree of similarity between the shape of compensatory motions and normal modes was estimated by calculating their root mean square inner product (RMSIP) (Supplementary Material). The magnitude of the RMSIP was generally high, with average values > 0.6 (Supplementary Table S7). This indicates that the double-force displacements have a good overlap with motions of the unperturbed essential space. The contributions of single normal modes to the RMSIP were generally non-uniform, with specific modes contributing more than the others (Fig. 2C). Interestingly, the modes featuring higher overlaps with the double-force displacements tend also to have a higher degree of collectivity (Brüschweiler, 1995) (orange line), indicating that the compensatory effects detected by DFS tend to involve the whole protein rather than be limited to the two perturbed sites. A detailed analysis of compensatory motions is presented as Supplementary Material (Supplementary Figs S4–S10).

### 3.2 Rescue pockets in p53

The results presented in the previous section indicate that DFS is able to identify rescue hotspots i.e. regions in the protein that are rich in rescue sites. This is particularly useful when screening a protein for sites that can be targeted by drugs. Indeed, targeting a rescue hotspot region has the advantage of maximizing the chances that a drug has a compensatory effect.

In order to detect possible rescue pockets i.e. ligand binding pockets that include also rescue sites, fpocket (Le Guilloux *et al.*, 2009) predictions were first run on the p53 structure. The resulting binding pockets were then defined as possible rescue pockets if at least 20% of their residues were rescue sites. A total of nine pockets were classified as rescue pockets on the basis of either experimental data or DFS predictions (Fig. 2D and Supplementary Table S8). Interestingly, the four pockets containing the largest number of experimental rescue sites (P3, P5, P7 and P11) were classified as rescue pockets also on the basis of DFS predictions (blue). Moreover, two of the DFS predicted pockets (P5 and P6) have been successfully targeted by drugs in the past (Boeckler *et al.*, 2008; Wassman *et al.*, 2013). In particular, drugs binding P5 have been shown to reactivate different p53 pathogenic mutants (Wassman *et al.*, 2013). The P5 pocket is composed for half of its residues by DFS rescue sites and it ranks second for rescue sites content (%RS_DFS_, Supplementary Table S8), so that it would have been screened as one of the best candidates even in the absence of experimental information and only using fpocket and DFS predictions.

### 3.3 Rescue sites and pathogenic mutations in p53

In the previous sections we described the performance of DFS in identifying potential rescue sites in p53, independently from the specific residues to be rescued. Here we want to test the ability of DFS to predict specific associations between rescue sites and residues involved in pathogenic mutations. To this aim, we compared the set of experimental and predicted rescue sites for each pathogenic mutation site PS in our p53 dataset (Supplementary Table S2). A DFS-predicted rescue site *j* was considered as potentially rescuing a PS *i* if the corresponding rescuability score $S_{\text{ij}}^{X}$ was > 0, with *X* either FF or FR.

**Fig. 3.**
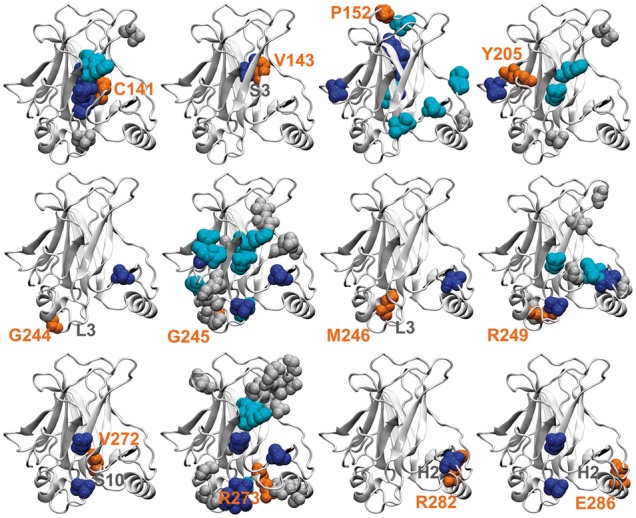
Detection of p53 rescue sites for selected pathogenic mutations. For each pathogenic mutation site PS (orange spheres), experimental rescue sites RS_exp_ are shown in blue (predicted by DFS as rescue sites for the specific PS site), cyan (within 4 Å from DFS sites) and grey (not predicted by DFS as associated to the PS site). Selected secondary structure elements are also labelled in grey (Color version of this figure is available at *Bioinformatics* online.)

For each PS position, we recorded the experimental rescue sites RS_exp_ that were correctly identified by DFS as compensatory residues for that position (blue spheres in Fig. 3 and Supplementary Table S9). At least one RS_exp_ residue was recovered in 14 out of 22 cases (64%), while for six PSs (V143, G244, M246, V272, R282 and E286), all the experimental rescue sites were identified. Overall, rescue sites of specific PS positions were predicted with an average sensitivity and specificity of 0.38 and 0.90, respectively (Supplementary Table S10). The average probability of finding an experimental compensatory residue in the DFS predictions for a given PS was 3.7 times the probability of a correct random guess (enrichment in Supplementary Table S10).

The clustering of rescue residues in specific regions means that even when some experimental rescue sites are missed, the relevant potential druggable regions with compensatory action can still be recovered. For this reason, we checked if among the RS_exp_ positions missed by DFS there were some in contact with DFS predictions (cyan spheres in Fig. 3). If these are considered, the number of PS positions for which at least one compensatory region is predicted by DFS increases to 19 (86% of PS positions, Supplementary Table S9).

The relationship between each PS position and the rescue pockets described in the previous section was also investigated, since it can give information on the regions of the protein surface to be targeted to reactivate a specific mutant. For each pocket we calculated the number of its residues that can rescue a specific PS position (Supplementary Table S11), using either predicted or experimental information. DFS detected at least one of the pockets classified as rescue on the basis of experimental sites for 16 out of 22 PS positions (73%, Supplementary Table S11). Position G245 is particularly interesting, since direct information on the binding sites of reactivating drugs is available for it. Indeed, mutations at this position have been shown to be rescued by different drugs binding at P5 (Wassman *et al.*, 2013). Remarkably, position G245 was predicted by DFS to be rescued by pockets P3, P5, P6 and P9, with P5 containing the second largest number of predicted G245 rescue sites after P3.

### 3.4 Prediction of rescue sites in CPD80

To further test DFS performance on different proteins, predictions from DFS were compared with putative rescue sites (RS_evol_) derived from evolutionary analyses (CPD80 dataset).

A good overall agreement between DFS predictions and RS_evol_ positions was found, with an average sensitivity of 0.33, specificity of 0.72 and accuracy of 0.67 (Table 1). In four cases [Cu-Zn superoxide dismutase (SOD), K-RAS GTPase (KRAS), transthyretin (TTR) and Interleukin 13 (IL13)] the performance was comparable or superior to that observed for p53, with sensitivity values ranging from 0.36 to 0.57, specificity values > 0.72 and accuracy values > 0.65.

**Fig. 4.**
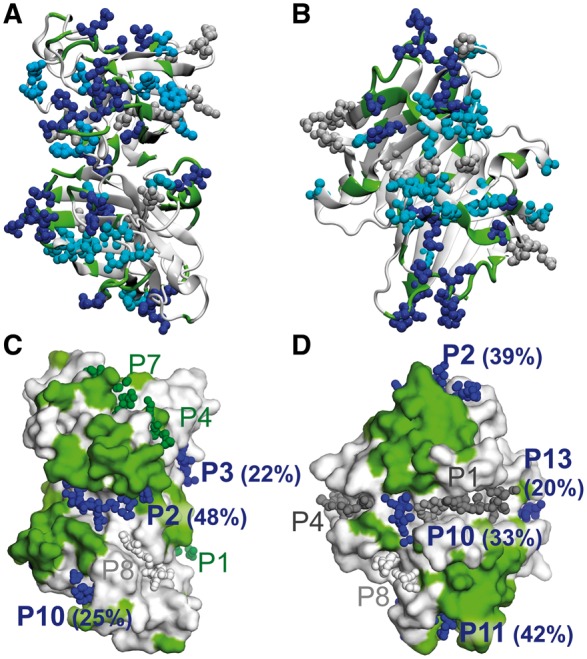
Comparison of evolutionary rescue sites and DFS-predicted rescue sites for SOD **(A,C)** and TTR **(B,D)**. (**A** and **B**) Evolutionary rescue sites RS_evol_ are mapped on the surface representation of the protein as spheres, while DFS-predicted rescue sites are shown as green cartoon. (**C** and **D**) Surface representation of the proteins, with surface DFS-predicted residues coloured in green. The α-sphere centres of the candidate pockets are shown as spheres. For all the panels the same colouring scheme is used as for Figure 2 (Color version of this figure is available at *Bioinformatics* online.)

Similarly to p53, the TTR and SOD proteins featured a non-uniform distribution of RS_evol_ residues (grey, cyan and blue spheres in Fig. 4A and B). Mapping the DFS predictions onto the structure shows that they parallel the RS_evol_ distribution, with DFS rescue sites (green cartoon) observed mostly in RS_evol_ hotspots. Accordingly, 44 and 36% of the RS_evol_ residues were identified by DFS as rescue sites for SOD and TTR, respectively (blue spheres in Fig. 4A and B), while an additional 35 and 38% (cyan spheres) was found in close proximity to DFS predicted sites (Supplementary Table S5).

The smaller sensitivity observed in the CPD80 other proteins can be justified on the basis of the nature of the reference RS_evol_ set. Indeed, this is not completely comparable to DFS predictions because (i) it can contain positions not necessarily related to compensatory mechanisms, (ii) multiple RS_evol_ residues might need to be mutated for a compensatory effect to be observed, while DFS considers only one second-site mutation at a time and (iii) analogously to p53, RS_evol_ can contain residues that use compensatory mechanisms not detectable by DFS because based on side chain mimicking or direct interactions with partners.

Possible binding pockets were predicted with fpocket for both SOD and TTR and their location was mapped onto the protein structures (spheres in Fig. 4C and D) together with the DFS-predicted rescue sites (green surface). The fraction of rescue sites in each pocket was calculated using either DFS predictions (%RS_DFS_, Supplementary Table S8) and evolutionary sites (%RS_evol_). Pockets with %RS ≥ 20 were classified as possible rescue pockets. Most of the rescue pockets detected on the basis of evolutionary data were classified as such also by using DFS predictions for both SOD (14 out of 15) and TTR (5 out of 8), further confirming the similarity in the spatial distribution of DFS and evolutionary rescue sites.

## 4 Discussion

In this article, we introduce the first general approach for the prediction of rescue sites in proteins. The DFS method aims at detecting compensatory effects that make use of the flexibility of the protein backbone to rescue native features affected by mutations. The only information required before the calculation is the native structure of the protein, while the effect of mutations is mimicked by the application of external forces. Differently from methods based on machine learning (Danziger *et al.*, 2009; Ramani and Jacob, 2013), no parameters in the model have been optimized against experimental rescue sites, so that DFS can be applied in principle to any protein with known 3D structure.

The types of compensatory effects that are detected by DFS can be mediated by either local backbone rearrangements around the first-site mutation (local changes) or changes of the overall backbone structure (global changes). The relative position of pathogenic/rescue residue pairs in p53 (Fig. 3) shows that rescue sites recovered by DFS can be either close to the residue they are rescuing (e.g. C141 or R282) or very far from it (e.g. G245 or E286), suggesting that long range communication between the two sites can be in place in some cases (Supplementary Fig. S11). Analysis of the distributions of the pathogenic site-rescue site distances shows that DFS can detect both short- and long-range pairs, even if with a larger proportion of short-range ones compared with the experimental data. At last, the decomposition of the compensatory motions in terms of normal modes shows that they tend to use modes with high collectivity indices, indicating that changes are not limited to the two sites but involve the whole protein.

The DFS method has been developed under the hypothesis that second-site mutations reverting any structural changes induced by a first-site mutation can potentially rescue also the protein function. This allows for the DFS method to be as general as possible, since it does not require any assumption on the specific relationship between protein function on one side and protein structure/dynamics on the other. Even if the DFS calculations performed in this work did not use any specific functional information, our predictions were found in striking agreement with the distribution of p53 sites known to rescue its function. It is also important to note that any information on the structure-dynamics-function relationship that might be available for specific cases can be easily introduced within the DFS framework by calculating additional rescuability indices based on specific functionally related properties (e.g. specific distances or angles) instead of all the atomic positions in the protein as used in this work.

Not all the types of compensatory mechanisms can be detected by DFS. These include compensatory effects based exclusively on (i) side chain mimicking and (ii) addition/removal of interactions with partners, as they both can occur with small or no impact on the protein backbone. Examples of both cases are present in p53. Indeed, the functional rescue of the R249S mutant by H168R is thought to be mainly due to side chain mimicking: the guanidinium group of R168 in the double mutant has a very similar position and orientation as the R249 guanidinium group in the wild type protein, thus rescuing its interactions and keeping the overall structure close to the native one (Joerger *et al.*, 2005). The second-site mutation T284R is instead thought to rescue p53 function by introducing additional interactions with DNA and compensating for the contacts that are lost in the cancer mutants R248Q or R273H (Wieczorek *et al.*, 1996). Similarly, the second-site mutation H178Y is thought to be directly involved in intermolecular interactions important for the formation of p53 tetramers (Otsuka *et al.*, 2007). The fact that positions 168, 178 and 284 do not rely on backbone motions as primary compensatory mechanism might explain why they are not predicted as rescue sites by DFS.

In a stricter performance assessment, DFS was tested to see if it was able not only to recover the residues most likely to have a compensatory effect, but also to predict the specific association between sites involved in pathogenic mutations and potential rescue sites. It was shown that DFS identified at least one experimental rescue site in 64% of the pathgenic sites in our p53 database, including some of the residues that are most commonly mutated in cancer such as G245 and R273 (Baronio *et al.*, 2010). On average, DFS recovered almost 40% of the rescue sites for a given pathogenic position. Even if not all the rescue sites were detected, potential compensatory pockets could still be identified. For example, the pathgenic site G245 was associated with pocket P5 even if not all its rescue sites were predicted. Remarkably, the G245S cancer mutant has been shown to be reactivated by different drugs binding p53 at P5 (Wassman *et al.*, 2013).

On the basis of the present calculations and performance data, the best protocol to follow when using DFS to identify potential drug binding targets for the reactivation of specific pathogenic mutants in a protein is:

1. Predict the rescue sites on the basis of their compensatory power. A percentile cutoff threshold of around the top 30% was shown to produce the best results for p53 and a good performance for other proteins in this study.
2. Identify potential binding sites for small molecules with a pocket detection method (Stank *et al.*, 2016; Volkamer and Rarey, 2014) and map onto them the rescue sites identified at step 1. Rank each pocket according to the fraction of its residues composed by rescue sites.
3. For each pathogenic mutation, identify which of the rescue sites found at step 1 are specifically predicted to rescue it on the basis of the rescuability scores. Rank the potential rescue pockets according to the number of these mutation-specific rescue sites included in them. Even if no rescue sites are detected for a given pathogenic site, the potential rescue pockets identified at step 2 might still be worth investigating. Indeed, DFS might have recovered the correct rescue sites but missed the connection with the specific pathogenic site.

The outcome of this protocol would be a ranked list of potential rescue pockets for each pathogenic site, to be further investigated to assess their druggability and to identify potential ligands.

In its current implementation, DFS predicts the position of candidate rescue mutations, but not the specific amino acidic changes that are required to compensate a given pathogenic mutation. This information might not be needed if the final goal is to identify possible regions to be targeted by compensatory drugs. However, generating a mutant that can be tested for functional rescue might be a direct way to validate DFS predictions. Following the same principle of the CPD database, sequences of close homologues could provide information on alternative amino acids that can be used at a given position in the sequence. These candidate mutations could then be tested for structural and functional rescue by *in silico* and *in vitro* assays.

As a final remark, it is important to note that within the DFS framework it is in principle possible to use different types of physical models to describe the protein dynamics and response to forces, such as a coarse grained model that includes C^β^ atoms (Micheletti *et al.*, 2004) or methods coupling Elastic Network Models with Brownian Dynamics to include non-linear responses to external forces and solvent effects (Lavery, 2009). Methods based on Molecular Dynamics simulations with atomistic force fields and enhanced sampling methods (Maximova *et al.*, 2016; Pandini and Fornili, 2016) could be also considered, especially when mutations are expected to produce large conformational changes that cannot be predicted from the normal modes of the native state only. Previous work has shown that covariance matrices from MD simulations can be effectively used in the context of the Linear Response Theory (LRT) (Kumar *et al.*, 2015).

In conclusion, we have shown that the DFS method can be effectively used to detect candidate rescue sites for pathogenic mutations using as input only the native structure of a protein. We believe that combined with drug design techniques, DFS can be a powerful tool to identify new drug targets for the development of ad hoc therapies of genetic disorders. Large-scale genome projects are currently generating a large amount of data on genome variability and its association with disease phenotypes, so that it can be foreseen that approaches based on rescuing disease-related mutations will become increasingly important in the future (Hwang and Sykes, 2015).

## Supplementary Material

Supplementary Data
