# Supplementary material for: In silico identification of rescue sites by double force scanning
Source: Bioinformatics. 2017 Aug 14;34(2):207–14. doi: 10.1093/bioinformatics/btx515 (PMC5860198; doi:10.1093/bioinformatics/btx515)
Supplement: Supplementary Data [file btx515_supplementarydata.zip › SupplementaryData_rev.pdf]

In silico identification of rescue sites by double force scanning

Matteo Tiberti, Alessandro Pandini, Franca Fraternali and Arianna Fornili

METHODS

Calculation of the rescuability index.

The compensatory effect was quantified by the rescuability index $\rho_{ij} = \left(d(u,p_i) - d(u,p_{ij})\right)/d(u,p_i)$, where *u* represents the native unperturbed structure, p_i and p_{ij} the structures upon application of the single and double forces, respectively, and *d(x,y)* is a distance function measuring the difference between structures *x* and *y*. Different choices are in principle possible for *d(x,y)*. The calculations presented in the paper were performed using the root mean square deviation between equivalent pair of distances (dRMSD)(Bordogna, et al., 2011) in order to describe changes affecting the overall structure. The rescuability of specific properties related to the protein function could be assessed by selecting function-related geometric variables (e.g. distances, angles, solvent accessible surfaces areas and cavity volumes) or dynamic properties (e.g. residue root mean square fluctuations and collective motions) for the calculation of *d*.

Forces with different orientations can produce different structural perturbations, making the value of ρ_{ij} dependent on the relative orientation of the forces applied at the two sites (Figure S1A). To take this into account, the following procedure was followed:

a) for a given first-site force vector $\mathbf{F_i}$, multiple orientations of the second-site force $\mathbf{F_j}$ are sampled and the rescuability index ρ_{ij} is calculated for each of them. The maximum value of ρ_{ij} (ρ_i^{MAX}) is then recorded.

b) the ρ_i^{MAX} values are recorded for different orientations of the first-site force $\mathbf{F_i}$ and an overall rescuability score S_{ij} is calculated as their average.

The final rescuability score S_{ij} can then be written as $S_{ij} = \langle \max_{\mathbf{F_j} \in \Omega} \rho_{ij} \rangle_{\mathbf{F_i} \in \mathrm{O}}$, where O and Ω indicate the set of $\mathbf{F_i}$ and $\mathbf{F_j}$ orientations, respectively. This procedure is based on the fact that, for a given pathogenic mutation, the second-site mutation can be optimised by choosing the amino acid change (mimicked by $\mathbf{F_j}$) that gives the maximum compensatory effect, while the amino acid change in the pathogenic mutation (mimicked by $\mathbf{F_i}$) cannot be modified. Averaging over $\mathbf{F_i}$ orientations is thus meant to provide an estimate of the average rescuability of mutations at *i*, independently from the specific amino acid change.

Scores for all pairwise comparisons were collected in two rescuability matrices $\mathbf{S^{FF}}$ and $\mathbf{S^{FR}}$, which were then combined in a unique score called the compensatory power *P*. First the number of residues rescued by a given second-site residue *j* (i.e. for which $S_{ij}^{FF/FR} > 0$) was calculated for both matrices and divided by the number of contacts of *j*, to give $P^{FF/FR}(j) = \sum_i (S_{ij}^{FF/FR} > 0)/N_C(j)$, where $N_C(j)$ is

the number of C^{α} atoms within r_c from *j*. The resulting P^{FF} and P^{FR} values were then rescaled from 0 to 1 and their average was used as the overall compensatory power *P*. Residues with higher *P* values have a higher potential of rescuing first-site residues according to FF and/or FR matrices.

The compensatory power *P* was used to predict experimental rescue sites in p53 and evolutionary rescue sites in CPD80 as described in the main text. Since the packing density can strongly influence the protein behaviour in ANM models, we investigated whether there was any relationship between the packing density around each residue, measured as the number of its contacts, and DFS predictions. Contacts were calculated using the position of Cα atoms, since it is the only measure of packing density that has an actual influence on the behaviour of the network model we used. For every residue of p53, we calculated the number of contacts as the number of Cα atoms within 15Å (ANM cutoff) and generated their distributions for the four classes of DFS predictions (true and false positives, true and false negatives). A comparison of these distributions with a Welch's t-test shows that they are not significantly different (the p-values between each pair range from 0.17 to 0.78), indicating that there is not a strong relationship between the packing density and the residues identified as compensatory by DFS. This is also consistent with the calculation of the compensatory power values used for the predictions, since they are normalised by the number of contacts.

Generation of force vectors

To avoid any bias, the sets of force vectors on each site need to be generated so that their spatial distribution around the point of application is as uniform as possible. Two different approaches were compared. In the first one (random approach) *N* force vectors were randomly generated sampling from a uniform distribution of the cosine of the angle between the force vector and a reference direction. In the second one (Fibonacci approach) the force vectors were generated according to the Fibonacci lattice, which is an approximate solution to the problem of finding *N* equally distant points on a sphere(Keinert, et al., 2015). The points of the Fibonacci lattice are arranged along a spiral, where the longitudinal angle between consecutive points is equal to the golden angle (~137.5°). In detail, the coordinates for each point *i,* with *i* ranging from 0 to *N* - 1*,* were generated in cylindrical coordinates as follows:

$$\rho_i = \sqrt{1 - z_i^2}$$

$$\varphi_i = i\theta$$

$$z_i = \left(1 - \frac{1}{N}\right)\left(1 - \frac{2i}{N-1}\right)$$

where ρ_i is the radial distance, φ_i the angular coordinate, z_i the height and θ is the golden angle ($\pi(3 - \sqrt{5})$). These coordinates were then converted to Cartesian coordinates and then scaled to the desired force vector length.

The Fibonacci method was more efficient than the random approach in terms of the number of force vectors N required to obtain a uniform distribution of force orientations (Figure S1B) and it was thus selected for the DFS implementation. Test calculations with this method showed that S_{ij} values converge for $N \geq 12$ (Figure S1C).

The relative magnitude of the forces applied on first sites i and second sites j was determined using two different schemes to reflect two possible scenarios. In the Fixed Force scheme (FF), the same magnitude was used for both $\mathbf{F_i}$ and $\mathbf{F_j}$, to mimic the situation where similar amino acidic changes occur at the two sites. In this case, the perturbation induced by the two forces applied singularly at the two sites can be very different (i.e. $d(u,p_i) \neq d(u,p_j)$) if there are large differences in their environment (e.g. number of contacts and flexibility). In the Fixed RMSD scheme (FR), force magnitudes were rescaled so that they produced structural perturbations of the same magnitude when applied singularly, i.e. $d(u,p_i) = d(u,p_j)$. This corresponds to the situation where the mutation at the second site can be finely tuned to compensate for differences in the environment at the two sites.

*Generation of the p53 and CPD80 databases***.**

A database of pathogenic and rescue mutations for p53 was generated from the available literature (Table S1). We collected the experimentally verified cases reported in the literature where a second site mutation was able to compensate the effect of a pathogenic mutation, therefore restoring partially or completely the ability of p53 to act as a transcription factor for one or more p53-activated promoter. In some cases, a pathogenic mutation was compensated by multiple mutations at the same time. Such mutations were all classified as rescue mutations. A dataset containing pathogenic (PS) and rescue sites (RS_{exp}) was then generated from the position of the pathogenic and rescue mutations in the p53 sequence (Table S2).

The CPD80 database (Table S3) was extracted from the Compensated Pathogenic Deviations (CPD) database(Barešić, et al., 2010), where a pathogenic mutation in a human protein is annotated as compensated if the mutated amino acid occurs in the wild type sequences of functionally equivalent proteins in other species. In CPD80, we considered only the database entries for which the percentage of identity between the human and functionally equivalent proteins was higher than 80%. Moreover, the following cases were excluded: i) proteins with largely incomplete experimental structure, to avoid the use of theoretical models, ii) proteins with a biological unit larger than 1000 residues or that belong to big multi-protein complexes, to reduce the computational cost, iii) non-globular proteins, including proteins with coiled-coil regions, long helices or disordered regions.

The experimental structures of CPD80 proteins used in this work (PDB IDs reported in Table S3) were selected according to the following procedure. For each protein, PDB IDs were derived by combining the UniProt/PDB cross-mappings available from the UniProt and PDB websites. The identified PDB entries were collected and all the biological unit structures as reported in the PDB were examined. Only the structures composed by the smallest number of chains of the protein among all its biological units were retained. The identified biological units contained the protein in dimeric form for Cu-Zn Superoxide dismutase and Transthyretin and in monomeric form for all the other proteins. The

final PDB structures of the CPD80 proteins were selected by matching as much as possible the following criteria: i) the proteins were not involved in macromolecular complexes with proteins other than themselves; ii) they were as complete and as close as possible to the wild-type sequence; iii) they were as devoid as possible of cofactors and extraneous molecules and iv) they had the best resolution available (if X-ray structures). In two cases (Antithrombin III and Thyroxine-binding globulin) missing inner solvent-exposed loops were identified and the MODLOOP webserver(Fiser and Sali, 2003) was used to model these short stretches of residues (28-31 and 353-358, respectively). Finally, the chosen PDB entries were filtered by retaining only the C^{α} atoms coordinates. Coordinates labelled with “A” were considered in case of alternate conformations.

For each CPD80 protein, the putative compensatory sites (evolutionary-related rescue sites or RS_{evol}) were detected by identifying the positions with different amino acids in pairwise alignments of the human and non-human homologs (Table S4). The alignments were generated with T-coffee (Poirot, et al., 2003) using default parameters. In the original CPD paper only local compensatory mutations were analysed by adopting a 8-Å cutoff on the distance between compensatory and compensated residues(Barešić, et al., 2010). To be able to compare them with DFS predictions, which take into account both short and long range effects, no distance cutoff was used here to determine the RS_{evol} positions in CPD80.

System setup and DFS parameters

ANM calculations were performed on the selected PDB structures of p53 (PDB ID: 1TSR, chain A) and of the CPD80 proteins (Table S3) with a distance cut-off r_c of 15 Å(Atilgan, et al., 2001) and a force constant γ of 0.1 kcal/mol/Å^2. Since we did not aim at reproducing specific magnitudes for the displacements (e.g. experimental values), the choice of γ was dictated by convenience of visualization (see below). The DFS was run on all the ordered pairs of residues for each system, generating a matrix of rescuability scores S_{ij} both for Fixed Force ($\mathbf{S}^{FF}$) and Fixed RMSD ($\mathbf{S}^{FR}$) . For both matrices, the application points of the forces (first and second sites) were excluded from the calculation of the structural distance function *d(x,y)* due to the large displacements produced by the forces at these sites. The number of force orientations *N* sampled for each site with the Fibonacci lattice method was set to 12 on the basis of preliminary convergence tests (see above). For the calculation of the Constant Force $\mathbf{S}^{FF}$ matrix, the magnitude of each force vector was set to 10.8 kcal/mol/Å for both sites to produce atomic displacements large enough (0.1 - 3.0 Å range) to be detected in the visualisation of the structures. Since the ANM model is purely harmonic, increasing the force magnitude and the γ value by the same scaling factor would not change the resulting displacements. Moreover, due to the linear relationship between forces and displacements, different values for the force magnitude would produce values of S_{ij}^{FF} that are in principle identical (except for deviations due to numerical errors). For the calculation of the $\mathbf{S}^{FR}$ matrix, the force magnitude was rescaled for each site and for each orientation to produce a *d* value of 1 Å when a single force was applied.

Determination of the reference threshold P_{cut}

As discussed in the main text, the threshold on the compensatory power P was determined only on DFS predictions of experimentally validated rescue sites (p53 dataset). We chose not to include CPD80 data in the calculation of P_{cut} as their relationship to actual rescue sites has not been experimentally demonstrated yet. In order to investigate the influence of the dataset on the final P_{cut} value, we performed a leave-one-out resampling test(Quenouille, 1956) on the dataset composed by p53 plus the 4 best performing CPD80 proteins Superoxide dismutase, KRas, Transthyretin and Interleukin-1 (Table 1). The remaining proteins were not considered because they had lower performances independently from the value of the threshold, suggesting either a lower reliability of the evolutionary data or an enrichment in rescue mechanisms not detectable by DFS compared to the best performing proteins. On this subset of five proteins, the leave-one-out resampling was performed at the residue level (equivalent to 1011 samples) and the percentile threshold was calculated as described in the main text (i.e. by minimising $D=(1 - \text{sensitivity})^2 + (1 - \text{specificity})^2$). The resulting threshold was 30.6±0.2%, which is very close to the value determined on p53 alone (28%).

Pocket detection and analysis

Candidate ligand binding pockets were identified running fpocket on the PDB structures of the proteins using default parameters. The fpocket algorithm is based on Voronoi tessellation and it has been shown to correctly identify known binding pockets with a true positive rate > 90%(Schmidtke, et al., 2010). The location of the pockets identified by fpocket is given by the position of the probe spheres used for their detection (α-spheres)(Le Guilloux, et al., 2009). The composition of a pocket is provided by fpocket as a list of atoms that are in contact with the centres of the α-spheres (Voronoi vertices). Pockets are ranked in order of decreasing fpocket score, which estimates the ability of the pocket to bind small ligands(Le Guilloux, et al., 2009). In this work, pockets with overall score < 0 or defined by buried α-spheres (cavities) were not taken into account. No further filters were applied on the pocket properties to be as comprehensive as possible. When used for drug design studies, promising rescue pockets should be further screened by including a description of their dynamics and by assessing their druggability. For example, the P5 pocket of p53 described in the main text ranks only fifth according to the fpocket overall score when the experimental structure is used, but it has been shown to significantly increase its volume and druggability during Molecular Dynamics (MD) simulations(Wassman, et al., 2013).

Analysis of rescued residues and compensatory motions

Positions predicted to be rescued by DFS rescue sites were clustered with the affinity propagation method(Frey and Dueck, 2007), using the negative squared euclidean distance between their C^{α} atoms as a measure for similarity. Each DFS rescue site *j* was first classified as FF or FR according to the relative magnitude of its compensatory power calculated from the rescuability matrices $\mathbf{S}^{FF}$ and $\mathbf{S}^{FR}$. The first sites *i* predicted to be rescued by *j* (i.e. for which the corresponding S_{ij}^{FF} or S_{ij}^{FR} is > 0)

were then collected and clustered using as input preference the value that maximises the average silhouette width(Rousseeuw, 1987).

The overlap between the displacements induced by the double force application (Δ**R**) and the normal modes of the unperturbed structures (**n)** was measured by the root mean square inner product (RMSIP)(Amadei, et al., 1999), calculated as $\sqrt{\sum_{l=1}^{m}|\Delta\mathbf{R}_c \cdot \mathbf{n}_l|^2}$. The first *m*=10 largest amplitude ANM normal modes $\mathbf{n}_l$ were considered (essential space). For each pair of residues *i* and *j*, RMSIP values were calculated by considering the displacement vectors $\Delta\mathbf{R_c}$ generated by the combinations of force orientations $\mathbf{F}_i$ and $\mathbf{F}_j$ with the maximum rescuability indices ρ_{ij} for both the FF and the FR scheme. For simplicity, these displacements are referred to as compensatory motions. Each of them was characterised by identifying its dominant normal mode $\mathbf{n}_{max}$, i.e. the normal mode with maximum inner product IP = $|\Delta\mathbf{R_c} \cdot \mathbf{n}|$.

To quantify the variability of compensatory motions used by a given second-site residue *j*, Shannon Entropies *H(j)* were calculated as $H(j) = -\sum_{l=1}^{m} P_l(j) log P_l(j)$, where $P_l(j)$ is the fraction of compensatory motions of *j* with dominant normal mode $\mathbf{n}_l$. RMSIP and H distributions were generated by collecting all the values associated to a given second-site residue *j* and assigning them to a specific class according to the compensatory power $P^{FF/FR}$ of *j*. $P^{FF/FR}$ classes were determined by partitioning the $P^{FF/FR}$ values in ten quantiles q_i, with q_i indicating $P^{FF/FR}$ values comprised between the (*i* -1)-th and *i*-th 10-quantile.

The degree of collectivity of a normal mode was estimated by calculating the collectivity index(Brüschweiler, 1995), which measures the fraction of C^α atoms affected by the mode.

ANALYSIS OF COMPENSATORY MOTIONS

The compensatory motions for a given first-site/second-site pair were defined as the double-force displacements associated with the largest values of the rescuability indices *ρ*, calculated using either FF or FR force magnitudes.

The relationship between normal modes and compensatory motions was investigated by analysing the distributions of the RMSIP values obtained for second-site residues of increasing compensatory power *P*. The results discussed here were obtained for p53 (Figure S4), but similar findings were observed in general for the other proteins (Figures S5-10). Two different behaviours were observed when using *P* values from FR and FF rescuability scores. Indeed, while an increase of the RMSIP values was observed for residues with high FR power (Figure S4C), the opposite trend was found when using FF values (Figure S4B). This indicates that the compensatory mechanisms detected by FR scores are based on an efficient use of collective motions that are already sampled by the unperturbed protein. On the other hand, FF compensatory motions tend to have a smaller degree of similarity with the protein essential space, indicating that FF rescue sites need to use additional motions to perform their compensatory action.

To investigate the range of variability in the type of compensatory motions used by a given second-site residue, we identified the dominant normal mode component of the motion for all the possible first sites and calculated the Shannon Entropy of the resulting distributions. Second-site residues with higher FF compensatory power turned out to have also higher Entropy values (Figure S4D), while the opposite behaviour was found when using FR scores (Figure S4E). This suggests that the rescue sites detected by FF scores tend to use a larger variety of motions than FR ones. This might be related to a higher heterogeneity of the residues rescued by FF rescue sites, as confirmed by their spatial distribution. Indeed, when residues rescued by a given rescue site were clustered according to their distance, a larger number of clusters were found for FF than FR rescue sites (Figure S4F).

To summarise, we found two different behaviours when describing the motions used by DFS-predicted rescue sites in terms of normal modes. Residues with high compensatory power can either use few motions that exploit efficiently the essential space of the unperturbed protein to rescue native-like structural features (FR sites) or use a multiplicity of motions, not necessarily from the essential space, to rescue multiple groups of first-site residues with different environments (FF sites). It is worth noting here that the main residues in the global suppressor motif (N235, N239 and S240) have higher FF contributions to the compensatory power (average FF contribution = 0.80) compared to the FR ones (average FR contribution = 0.23, p-value from a Welch t-test comparison of the FF and FR distributions = 0.02), suggesting that their ability to rescue a large number of residues might be related to an efficient use of different compensatory motions. Correspondingly, their average FF Shannon Entropy (2.52) is larger than the average calculated over all the residues (2.40).

COMPARISON OF DFS PREDICTIONS FROM DIFFERENT P53 STRUCTURES

In order to assess the stability of the DFS compensatory power for small structural changes, we compared the results described in the main text for the DNA-free structure of p53 (PDB ID: 1TSR, chain A) with those obtained on the DNA-bound state (PDB ID: 4HJE, chain A). In both cases, residues from 94 to 289 were considered.

DNA binding induces small structural changes and the two conformations differ by an RMSD on C_α atoms of 1.1 Å. The compensatory power profiles are correspondingly very similar (Figure S3), with a Pearson correlation coefficient of 0.89. The sets of top 28% residues by compensatory power (defined as the DFS rescue sites) are mostly superimposable, with analogous overall features and localisation on the protein structure. The region of the profiles that behaves most differently (residues 160-175) corresponds to a part of the protein that is close to the flexible N-terminal tail, which has a significantly different conformation in the two structures.

The overall DFS performance for the DNA-bound state was similar to the DNA-free one, with a sensitivity of 0.388, specificity of 0.755 and accuracy of 0.663. A slight reduction was observed in the number of experimentally-determined rescue residues detected by DFS (19 instead of 23 in the DNA-free state), but 3 of the 4 missed predictions were still in contact with DFS-predicted rescue sites,

meaning that these differences do not affect our ability to identify the regions of the protein with high compensatory potential also in the DNA-bound conformation.

REFERENCES

Amadei, A., Ceruso, M.A. and Di Nola, A. On the convergence of the conformational coordinates basis set obtained by the essential dynamics analysis of proteins' molecular dynamics simulations. *Proteins* 1999;36(4):419-424.

Atilgan, A.R., *et al.* Anisotropy of fluctuation dynamics of proteins with an elastic network model. *Biophys. J.* 2001;80(1):505-515.

Barešić, A., *et al.* Compensated pathogenic deviations: analysis of structural effects. *J. Mol. Biol.* 2010;396(1):19-30.

Bordogna, A., Pandini, A. and Bonati, L. Predicting the accuracy of protein-ligand docking on homology models. *J. Comp. Chem.* 2011;32(1):81-98.

Brüschweiler, R. Collective protein dynamics and nuclear spin relaxation. *J. Chem. Phys.* 1995;102(8):3396-3403.

Fiser, A. and Sali, A. ModLoop: automated modeling of loops in protein structures. *Bioinformatics* 2003;19(18):2500-2501.

Frey, B.J. and Dueck, D. Clustering by passing messages between data points. *Science* 2007;315(5814):972-976.

Keinert, B., *et al.* Spherical fibonacci mapping. *ACM Trans Graph* 2015;34(6):1-7.

Le Guilloux, V., Schmidtke, P. and Tufféry, P. Fpocket: An open source platform for ligand pocket detection. *BMC Bioinformatics* 2009;10(1):168-111.

Poirot, O., O'Toole, E. and Notredame, C. Tcoffee@igs: A web server for computing, evaluating and combining multiple sequence alignments. *Nucleic Acids Res.* 2003;31(13):3503-3506.

Quenouille, M.H. Notes on bias in estimation. *Biometrika* 1956;43:353-360.

Rousseeuw, P.J. Silhouettes - a Graphical Aid to the Interpretation and Validation of Cluster-Analysis. *J. Comput. Appl. Math.* 1987;20:53-65.

Schmidtke, P., *et al.* fpocket: online tools for protein ensemble pocket detection and tracking. *Nucleic Acids Res.* 2010;38(Web Server):W582-W589.

Wassman, C.D., *et al.* Computational identification of a transiently open L1/S3 pocket for reactivation of mutant p53. *Nat Commun* 2013;4:1407-1409.

TABLES

Table S1. Dataset of pathogenic and rescue mutations of p53 (available as TableS1.xls).

Table S2. Experimental rescue sites in p53 for each pathogenic site.

PS	RS_{exp}[a]
N131	N239
C141	D228,I232,H233,Y234,N235,Y236,N239,S240
V143	N268
P152	Q100,K101,Y103,S106,H115,T118,T123,L137,D207
V157	Y234,N235,N239
R158	Q100,Q104,L114,H115,L201,D207,E224,S227,D228,H233,Y234, N235,N239,S240
Y163	H233,N235,N239,S240
V173	S227,D228,T231,N235,N239,S240
P177	V122
Y205	D207,D228,H233,N235,N239
Y220	D228,T230,Y234,N235,N239,S240
G244	T123
G245	F113,L114,T123,A159,V172,H178,C182,S183,D184,D186,G187, L188,A189,T230,T231,H233,Y234,N235,N239,S240
M246	V122
R248	H115,T284
R249	T118,V122,T123,C124,K139,H168,S227,T231,N235,N239
L252	V122
V272	N235,N239
R273	Q100,Q104,T123,Q144,H178,S183,E224,G226,D228,C229,T230, I232,H233,N235,N239,S240,S241,C242,N263,D281,T284,E285
G279	V122,C124,H168
R282	V122
E286	N235,N239

[a]A rescue site was associated with a given pathogenic site if it was found mutated in at least one experimental rescue mutant for that site (see Table S1 for a detailed list of rescue mutants).

Table S3. Proteins in the CPD80 dataset.

Uniprot ID	Protein Name	Short Name	PDB ID	nres[a]	seq. ide.[b]
P00441	Cu-Zn Superoxide dismutase[c]	SOD	2C9U	306	83
P01008	Alpha Antithrombin III	AT-III	1E03	427	87
P01009	Alpha-1-Antitrypsin	AAT	3CWL	372	96
P01116	K-RAS GTPase	KRAS	4LPK	166	89
P02766	Transthyretin[c]	TTR	4TLT	230	82
P02768	Serum albumin	ALB	4L8U	583	82
P05543	Thryoxine-binding globulin	TBG	4X30	378	82
P18440	Arylamine N-acetyltransferase 1	AAC1	2PQT	295	83
P35225	Interleukin 13	IL13	1IK0	113	95
P51580	Thiopurine S-methyltransferase	TPMT	2BZG	229	81

[a] Number of residues in the Biological Unit (BU)

[b] Minimum sequence identity between human and non-human homologues in CPD80

[c] Homodimer in the BU

Table S4. Evolutionary rescue sites RS_{evol} for each protein in CPD80 (available as TableS4.xls).

Table S5. Percentage of p53 rescue sites predicted by DFS.

Protein	nRS_{exp} (DFS)[a]	nRS_{exp} (tot)[b]	$\%RS_{exp}$ (DFS)[c]	nRS_{exp} (contact)[d]	$\%RS_{exp}$ (contact)[e]	$\%RS_{exp}$ (DFS + contact)[f]
p53	23	49	47	17	35	82
SOD	27	62	44	22	35	79
AT-III	15	51	29	23	45	75
AAT	3	14	21	2	14	36
KRAS	4	7	57	2	29	86
TTR	21	58	36	22	38	74
ALB	30	104	29	32	31	60
TBG	18	73	25	29	40	64
AAC1	13	49	27	14	29	55
IL13	2	5	40	2	40	80
TPMT	13	61	21	28	46	67

[a]Number of RS_{exp} sites predicted by DFS

[b]Total number of RS_{exp} sites

[c]Percentage of RS_{exp} sites predicted by DFS

[d]Number of RS_{exp} sites in contact (non-hydrogen atoms within 4 Å) with DFS predictions

[e]Percentage of RS_{exp} sites in contact with DFS predictions

[f]Percentage of RS_{exp} sites predicted by DFS or in contact with DFS predictions

Table S6. Experimental and predicted rescue sites in p53.

RS type	resid
RS_{exp} (blue)[a]	K101,Y103,Q104,F113,V122,T123,C124,K139,D186,L188,A189, L201,D207,D228,C229,Y234,N235,Y236,N239,S241,C242,N263, N268
RS_{exp} (cyan)[b]	Q100,S106,L114,H115,L137,Q144,A159,H168,V172,D184,G187, E224,S227,T230,I232,H233,S240
RS_{exp} (grey)[c]	T118,H178,C182,S183,G226,T231,D281,T284,E285
RS_{DFS}[d]	K101,T102,Y103,Q104,G105,R110,G112,F113,S121,V122,T123, C124,K139,T140,C141,P152,P153,G154,I162,V173,R174,D186,L 188,A189,I195,R196,V197,E198,L201,D207,D228,C229,Y234,N2 35,Y236,C238,N239,S241,C242,M243,G244,G245,M246,N247,R 248,R249,D259,S260,S261,N263,R267,N268,S269,F270,E271

[a]Experimental rescue sites predicted by DFS.

[b]Experimental rescue sites in contact with DFS-predicted rescue sites.

[c]Experimental rescue sites not predicted and not in contact with DFS-predicted rescue sites.

[d]DFS-predicted rescue sites.

Table S7. Average overlap (RMSIP) between compensatory motions and essential space for p53 and CPD80 proteins.

Protein	<RMSIP>	
	FF[a]	FR[b]
p53	0.57	0.54
SOD	0.77	0.73
KRAS	0.50	0.46
TTR	0.66	0.62
AAC1	0.49	0.46
TPMT	0.52	0.49
AT-III	0.61	0.56
AAT	0.68	0.64
ALB	0.92	0.89
TBG	0.69	0.65
IL13	0.77	0.67
Average	0.65	0.61

[a]Value calculated over the FF $\Delta\mathbf{R}_c$ displacements of all first-site/second-site pairs

[b]Value calculated over the FR $\Delta\mathbf{R}_c$ displacements of all first-site/second-site pairs

Table S8. Percentage of rescue residues in the pockets predicted for p53, SOD and TTR.

Pocket[a]	$\%RS_{DFS}$[b]	$\%RS_{exp/evol}$[c]
	p53	
P1	15	12
P2	16	26
**P3**	**36**	**29**
**P5**	**50**	**42**
P6	20	0
**P7**	**20**	**40**
P9	38	13
P10	18	27
**P11**	**64**	**36**
P12	33	11
	SOD	
P1	22	3
**P2**	**48**	**24**
**P3**	**22**	**33**
**P4**	**38**	**38**
**P5**	**33**	**25**
**P6**	**63**	**38**
P7	43	14
P8	0	12
P9	46	8
**P10**	**25**	**42**
P11	15	0
**P12**	**85**	**54**
**P13**	**33**	**42**
**P14**	**64**	**55**
**P15**	**40**	**20**
**P16**	**54**	**31**
P17	0	10
**P18**	**33**	**25**
**P19**	**33**	**56**
**P20**	**50**	**30**
P21	11	22
	TTR	
P1	14	52
**P2**	**39**	**46**
P4	7	36
P5	44	0
P6	17	42
P7	22	0
P8	15	8
P9	8	0
**P10**	**33**	**50**
**P11**	**42**	**25**
**P12**	**40**	**40**
**P13**	**20**	**40**
P15	38	0

[a]Pockets ranked according to the fpocket score. Pockets with both $\%RS_{DFS}$ and $\%RS_{exp/evol}$ >= 20 are highlighted in bold. Pockets with a negative fpocket score or buried alpha spheres were not considered.

[b]Percentage of pocket residues classified as rescue sites according to DFS predictions.

[c]Percentage of pocket residues classified as rescue sites according to experimental (p53) and evolutionary (SOD and TTR) data.

Table S9. Experimental p53 rescue sites (RS_{exp}) predicted by DFS for each pathogenic site (PS).

PS	Region[a]	%RS_{exp} (DFS)[b]	RS_{exp} (DFS)[c]	%RS_{exp} (DFS+contact)[d]	RS_{exp} (DFS+contact)[e]
131	S2'	0	-	100	239
141	S3	38	234, 235, 236	63	232, 233, 234, 235, 236
143	S3	100	268	100	268
152	S3-S4	33	101, 103, 207	89	100, 101, 103, 106, 115, 123, 137, 207
157	S4	0	-	0	-
158	S4	0	-	21	100, 233, 234
163	S4	25	239	75	235, 239, 240
173	L2	0	-	50	235, 239, 240
177	L2	0	-	100	122
205	S6	20	207	60	207, 233, 235
220	S7-S8	0	-	17	235
244	L3	100	123	100	123
245	L3	15	123, 189, 239	55	123, 172, 186, 187, 188, 189, 233, 234, 235, 239, 240
246	L3	100	122	100	122
248	L3	0	-	0	-
249	L3	20	122, 239	50	122, 123, 124, 235, 239
252	S9	0	-	0	-
272	L-S-H motif (S10)	100	235, 239	100	235, 239
273	L-S-H motif (S10)	23	123, 235, 239, 241, 242	36	123, 232, 233, 235, 239, 240, 241, 242
279	L-S-H motif (H2)	67	122, 124	100	122, 124, 168
282	L-S-H motif (H2)	100	122	100	122
286	L-S-H motif (H2)	100	235, 239	100	235, 239

[a]Secondary structure element where PS is located

[b]Percentage of RS_{exp} residues predicted by DFS as rescuing a specific PS

[c]Indices of the RS_{exp} residues predicted by DFS as rescuing a specific PS

[d]Percentage of RS_{exp} residues predicted by DFS as rescuing a specific PS or in contact (non-hydrogen atoms within 4 Å) with DFS predictions

[e]Indices of RS_{exp} residues predicted by DFS as rescuing a specific PS or in contact (non-hydrogen atoms within 4 Å) with DFS predictions

Table S10. DFS performance in predicting rescue sites of specific pathogenic sites of p53.

PS[a]	sensitivity[a]	specificity[b]	fall-out[c]	enrichment[d]
131	0.000	0.918	0.082	0.000
141	0.375	0.930	0.070	4.570
143	1.000	0.938	0.062	15.000
152	0.333	0.806	0.194	1.667
157	0.000	0.964	0.036	0.000
158	0.000	0.950	0.050	0.000
163	0.250	0.927	0.073	3.250
173	0.000	0.931	0.069	0.000
177	0.000	0.835	0.165	0.000
205	0.200	0.963	0.037	4.875
220	0.000	0.958	0.042	0.000
244	1.000	0.845	0.155	6.290
245	0.150	0.891	0.109	1.330
246	1.000	0.902	0.098	9.750
248	0.000	0.891	0.109	0.000
249	0.200	0.919	0.081	2.294
252	0.000	0.948	0.052	0.000
272	1.000	0.933	0.067	13.000
273	0.227	0.936	0.064	2.770
279	0.667	0.849	0.151	4.194
282	1.000	0.876	0.124	7.800
286	1.000	0.736	0.264	3.679
average	0.382	0.902	0.098	3.658

[a] Residue index (pathogenic site).

[b] Sensitivity or true positive rate = TP/(TP + FN), with TP = true positives, FN = false negatives

[c] Specificity or true negative rate = TN/(TN + FP), with TN = true negatives, FP = false positives

[d] Fall-out or false positive rate = FP/(TN + FP)

[e] Enrichment in experimental rescue sites of DFS predictions = P_{DFS} / P_{random}, where P_{DFS} = TP / (TP + FP) and P_{random} = (TP + FN) / (nres - 1), with nres = total number of residues.

Table S11. Association between pathogenic sites (PS) and rescue pockets in p53.

PS[a]	P2[b]		**P3**		**P5**		P6		**P7**	
	DFS[c]	Exp[d]	**DFS**	**Exp**	**DFS**	**Exp**	DFS	Exp	**DFS**	**Exp**
131	0	0	**1**	**1**	**3**	**0**	0	0	**0**	**0**
141	0	1	**1**	**2**	**4**	**0**	0	0	**1**	**2**
143	0	0	**0**	**0**	**1**	**0**	0	0	**1**	**0**
152	2	0	**1**	**0**	**3**	**1**	2	0	**2**	**0**
157	0	0	**0**	**1**	**0**	**0**	0	0	**0**	**0**
158	0	2	**0**	**2**	**0**	**1**	0	0	**1**	**2**
163	0	0	**4**	**2**	**0**	**0**	0	0	**0**	**1**
173	0	2	**0**	**2**	**0**	**0**	0	0	**0**	**1**
177	0	0	**5**	**0**	**3**	**1**	0	0	**2**	**0**
205	0	1	**0**	**1**	**0**	**0**	0	0	**1**	**1**
220	0	2	**0**	**2**	**0**	**0**	0	0	**0**	**0**
244	0	0	**5**	**0**	**4**	**1**	0	0	**2**	**0**
245	0	1	**5**	**2**	**3**	**3**	0	0	**0**	**2**
246	0	0	**5**	**0**	**2**	**1**	0	0	**0**	**0**
248	0	0	**4**	**0**	**3**	**0**	0	0	**0**	**0**
249	0	1	**4**	**1**	**0**	**3**	0	0	**0**	**1**
252	0	0	**1**	**0**	**0**	**1**	0	0	**0**	**0**
272	0	0	**2**	**1**	**0**	**0**	0	0	**0**	**0**
273	0	4	**2**	**4**	**0**	**1**	0	0	**0**	**3**
279	0	0	**5**	**0**	**6**	**2**	0	0	**1**	**0**
282	0	0	**4**	**0**	**4**	**1**	0	0	**0**	**0**
286	2	0	**5**	**1**	**6**	**0**	3	0	**2**	**0**

[a]Residue index (pathogenic site).

[b]Pocket label from fpocket, see legend of Table S8.

[c]Number of sites in the pocket that are predicted by DFS to rescue a given PS.

[d]Number of sites in the pocket that are experimentally known to rescue a given PS.

Table S11 - continued

PS	P8		P9		P10		**P11**		P12	
	DFS	Exp	DFS	Exp	DFS	Exp	**DFS**	**Exp**	DFS	Exp
131	0	0	3	0	2	0	**1**	**0**	1	0
141	0	0	3	0	1	0	**3**	**1**	0	0
143	0	0	2	0	2	0	**2**	**0**	0	0
152	0	1	3	1	2	1	**6**	**0**	1	0
157	0	0	0	0	0	0	**0**	**1**	0	0
158	0	0	0	1	0	2	**1**	**2**	0	0
163	0	0	3	0	0	0	**0**	**1**	2	0
173	0	0	0	0	0	0	**0**	**1**	2	0
177	0	1	2	0	0	0	**7**	**0**	3	0
205	0	0	0	0	0	0	**3**	**1**	0	0
220	0	0	0	0	0	0	**0**	**1**	0	0
244	1	0	2	0	0	0	**6**	**0**	2	0
245	0	0	1	0	0	2	**0**	**3**	2	1
246	0	1	1	0	0	0	**0**	**0**	3	0
248	0	1	1	0	0	1	**0**	**0**	3	0
249	0	2	2	0	0	0	**0**	**1**	2	0
252	0	1	3	0	0	0	**1**	**0**	0	0
272	0	0	2	0	0	0	**1**	**1**	0	0
273	0	1	1	1	0	0	**1**	**1**	0	0
279	1	1	3	0	1	0	**4**	**0**	2	0
282	1	1	3	0	0	0	**3**	**0**	2	0
286	1	0	3	0	2	0	**7**	**1**	3	0

FIGURES

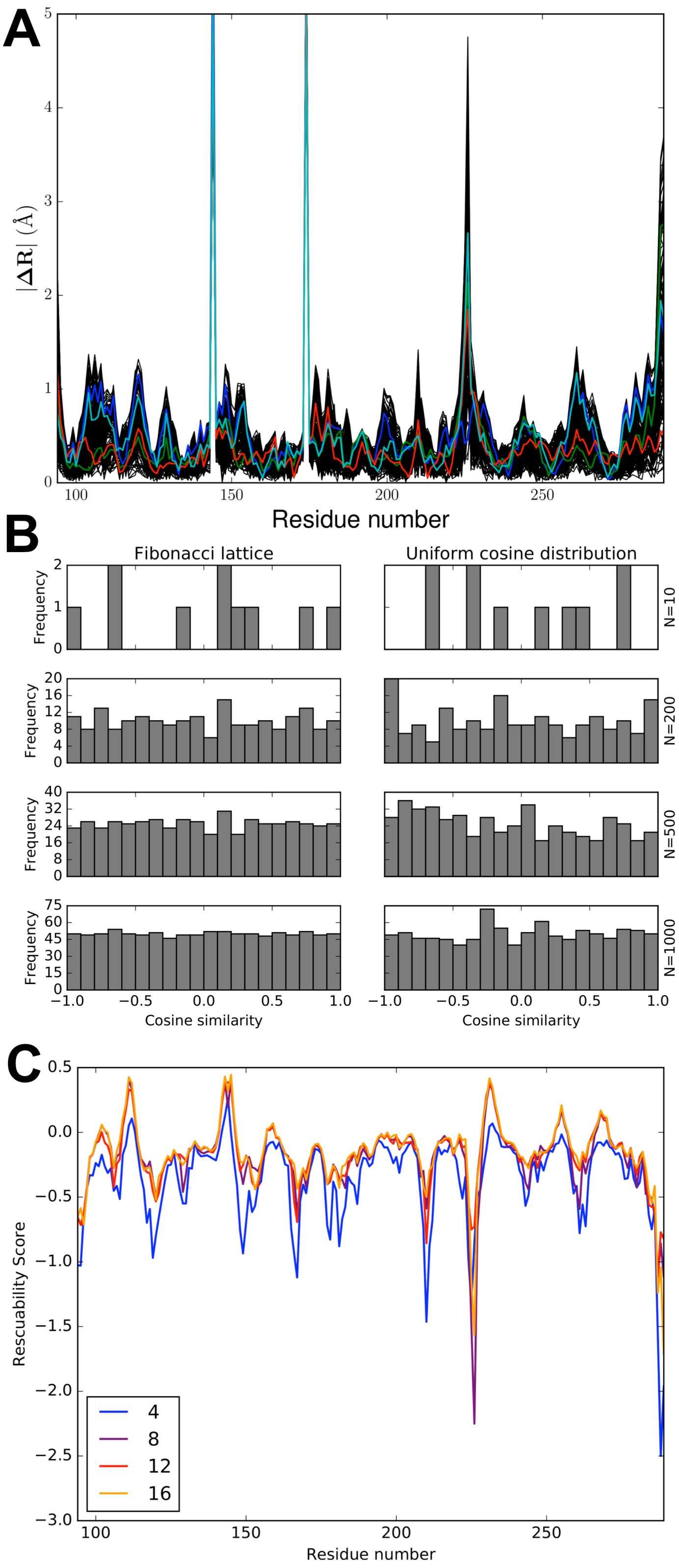

Figure S1. (A) Magnitude of p53 atomic displacements resulting from different combinations of force directions applied at residues 144 and 174. Forces were applied according to the DFS FF protocol, using 12 different Fibonacci-lattice orientations per site (for a total of 144 combinations). The

displacement profiles from each pair of forces are represented as black lines. Representative profiles were obtained by clustering all the displacement profiles with the Affinity Propagation method (coloured lines). Values corresponding to the first and second sites are ~ 6 Å. b) Distributions of cosine similarity values calculated between a reference vector (1, 1, 1) and sets of vectors generated according to the Fibonacci (left panels) and the random (right panels) approach. An increasing number of samples was used (N=10, 200, 500 and 1000 from top to bottom). c) Profiles of FF rescuability scores S_{ij} calculated for a specific first site (residue 144) using an increasing number of force orientations per site.

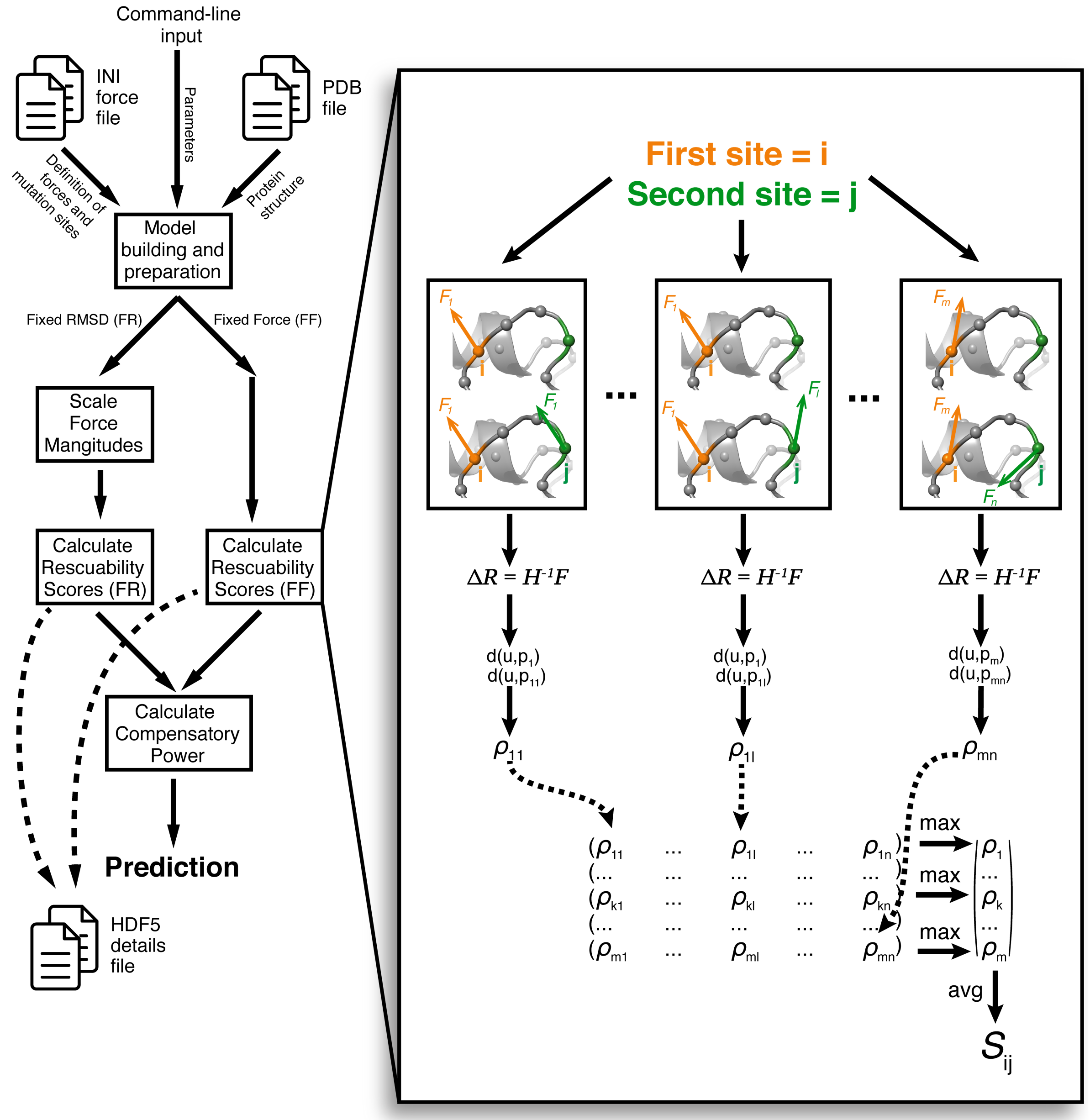

Figure S2. Overview of the DFS method. A high-level workflow is shown on the left. First, the protein structure and some custom parameters (e.g. subsets of sites to be analysed instead of the whole protein) are read as input. DFS is then run both in FR and the FF mode. Intermediate information from the calculation can be saved to a HDF5 database file with a level of detail set by the user. The resulting rescuability score matrices **S** are then used to calculate the compensatory power *P*. The calculation of the score matrices is illustrated in detail on the left. Forces are applied either to the single site *i* or to both sites *i* and *j* and the resulting atomic displacements are calculated. The rescuability index ρ is calculated for every pair of force orientations and these are combined as shown to get the final rescuability score S_{ij}.

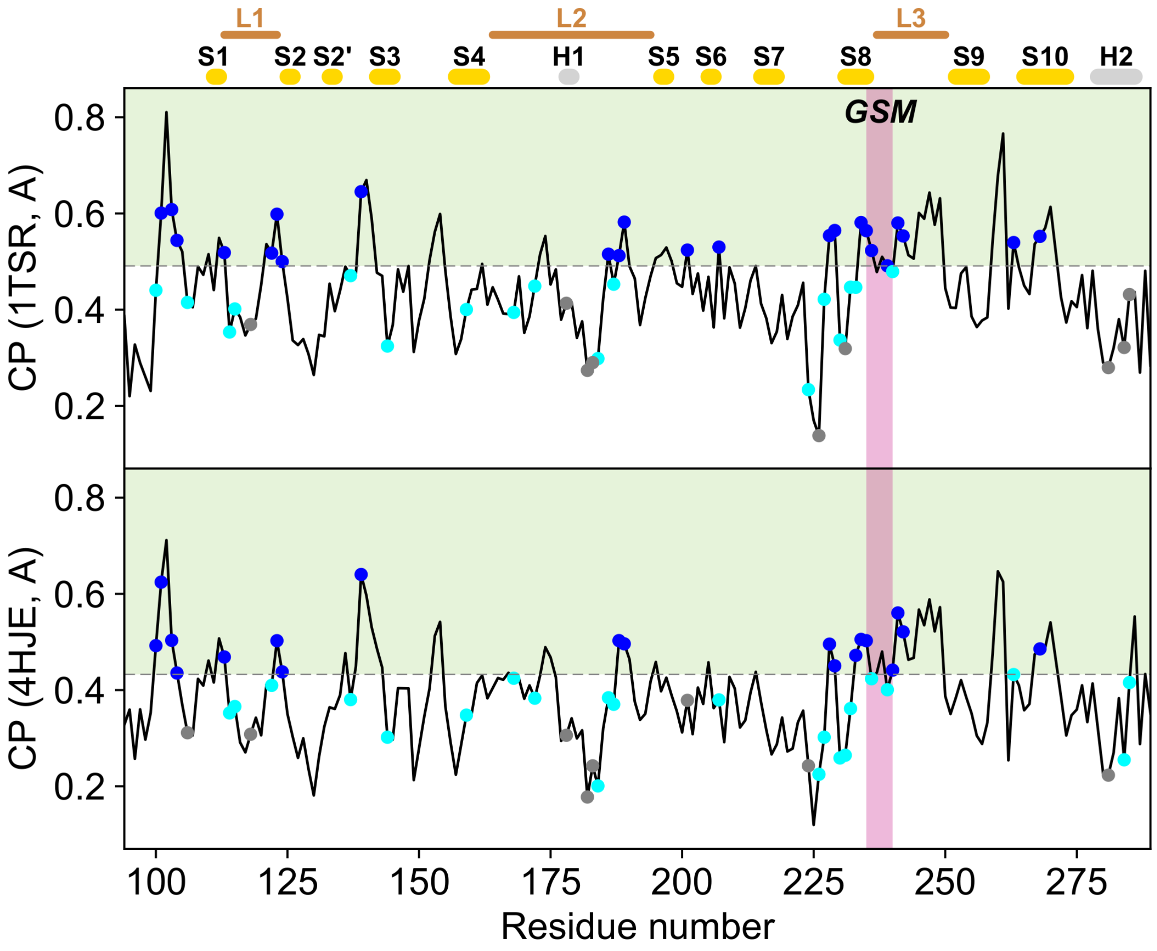

Figure S3. Plot of DFS compensatory power *P* for the DNA-free (1TSR, A) and DNA-bound (4HJE, A) structures of p53. The threshold P_{cut} used for the definition of DFS rescue sites is represented with a dashed grey line. Experimental rescue sites RS_{exp} are indicated with dots coloured in blue (predicted by DFS), cyan (within 4 Å from DFS rescue sites) and grey (not predicted). Secondary structure elements are indicated with gold (strands) and grey (helices) blocks. Loops L1-3 are indicated with brown lines and the position of the global suppressor motif (GSM) is shaded in magenta.

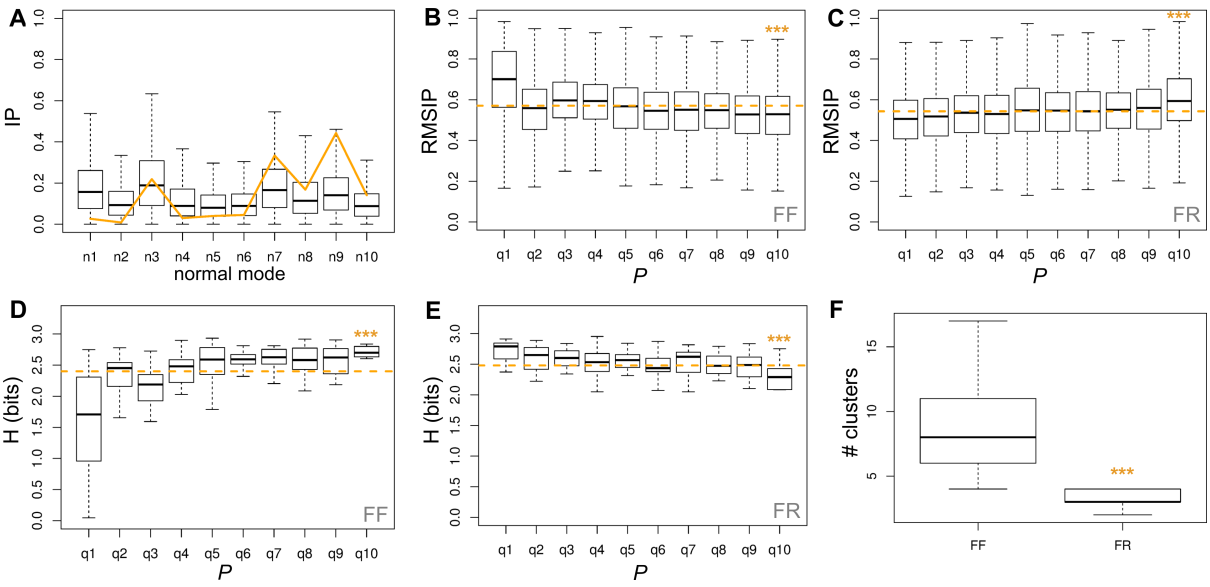

Figure S4. Analysis of compensatory motions in p53. (A) Distributions of inner products calculated between the FF compensatory motions $\Delta\mathbf{R}_c$ and each of the first 10 normal modes represented as boxplots. The collectivity index of each normal mode is reported in orange. (B/C) Distributions of RMSIP values calculated between the first 10 normal modes and the FF (B) and FR (C) compensatory motions $\Delta\mathbf{R}_c$, classified according to the compensatory power *P* of the second-site residue. *P* values are partitioned in ten classes q_i, with q_i indicating *P* values comprised between the (*i* -1)-th and *i*-th 10-quantile. A t-test comparison was performed between the first (q_1) and last (q_{10}) distributions, which are found different with a level of significance indicated by the orange stars (*** p-value <0.001). (D/E) Distributions of dominant normal-mode Shannon Entropy values for the FF (D) and FR (E) compensatory motions $\Delta\mathbf{R}_c$, classified according to the compensatory power *P*. (F) Distribution of the number of clusters of rescued residues for FF and FR rescue sites.

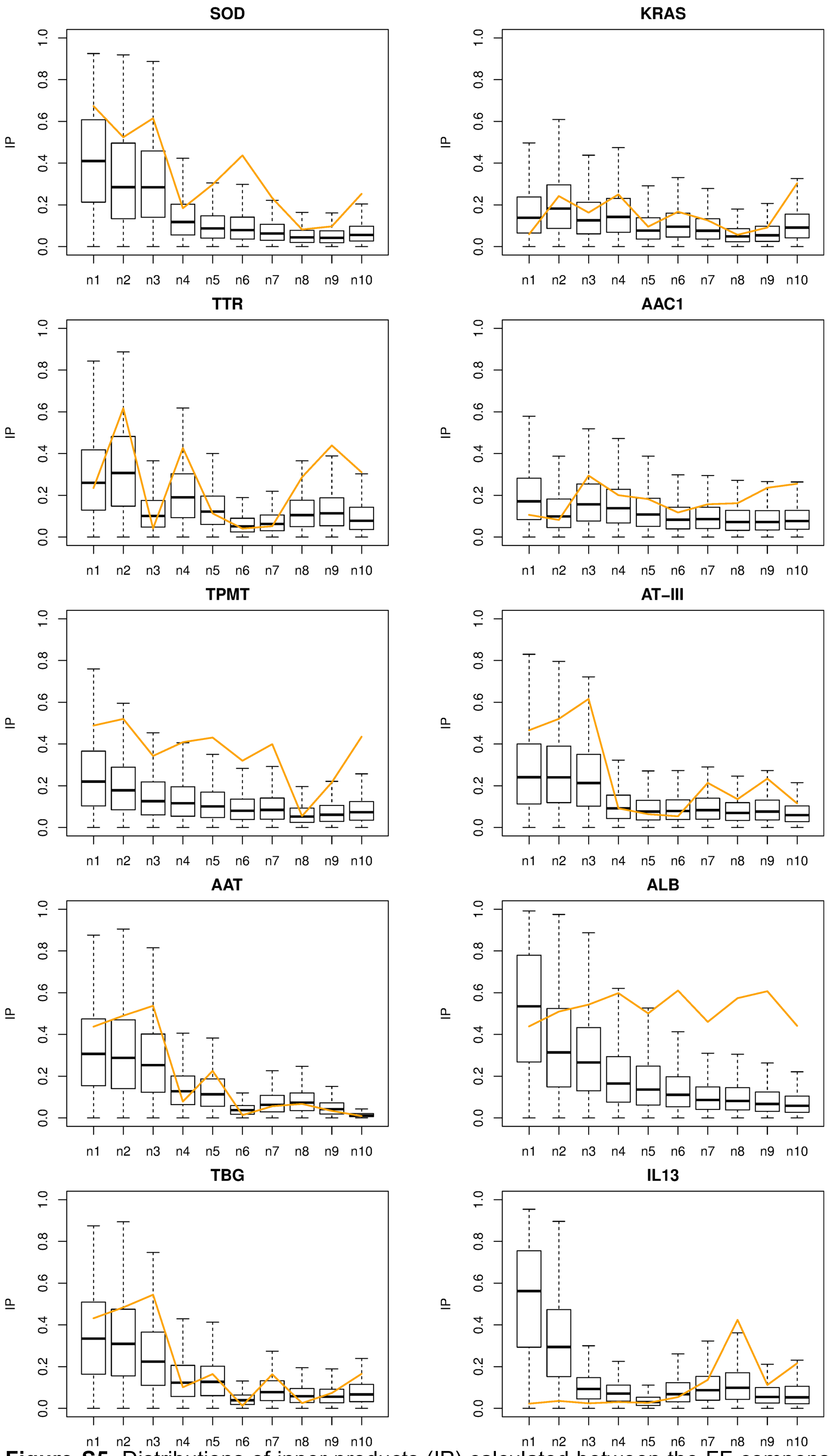

Figure S5. Distributions of inner products (IP) calculated between the FF compensatory motions $\Delta \mathbf{R}_c$ and each the first 10 normal modes of CPD80 proteins represented as boxplots. The collectivity index of each normal mode is reported in orange.

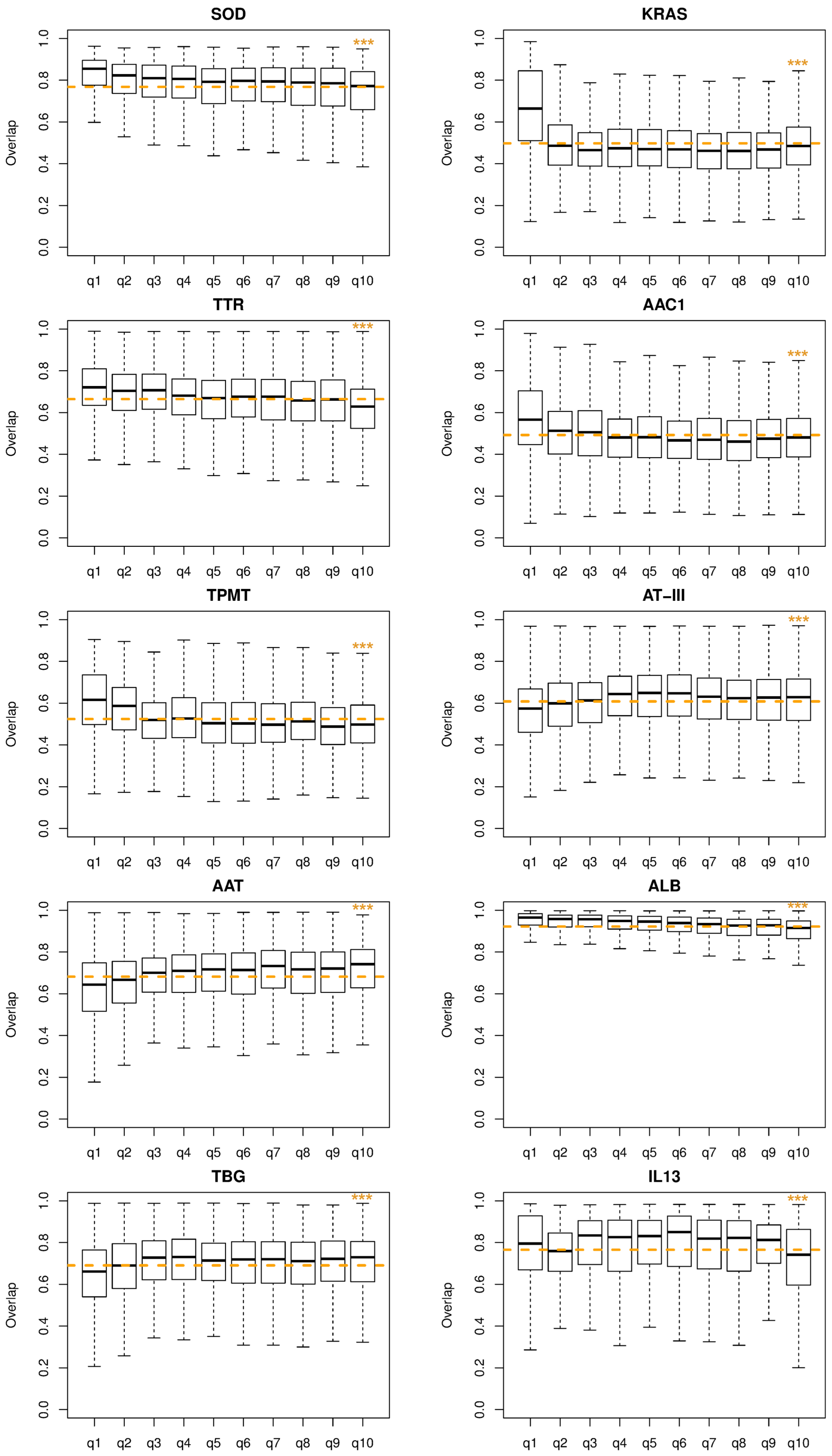

Figure S6. Distributions of RMSIP values calculated between the first 10 normal modes and the FF compensatory motions $\Delta\mathbf{R}_c$ of CPD80 proteins, classified according to the compensatory power *P* of the second-site residues. *P* values are partitioned in ten classes q_i, with q_i indicating *P* values comprised between the (*i* -1)-th and *i*-th 10-quantile. A t-test comparison was performed between the first (q_1) and last (q_{10}) RMSIP distributions, which are found different with a level of significance indicated by the orange stars (*** p-value <0.001).

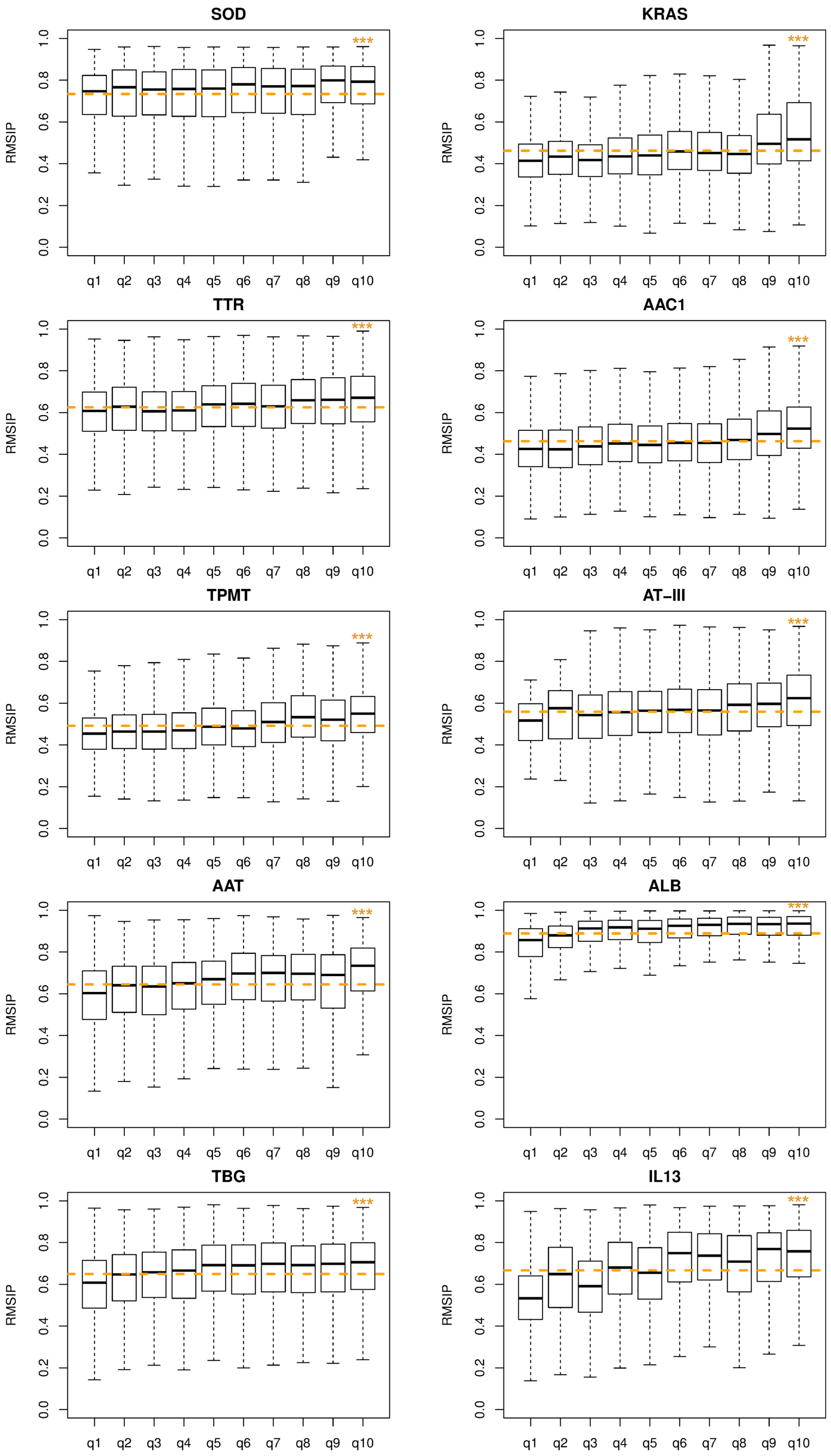

Figure S7. Distributions of RMSIP values calculated between the first 10 normal modes and the FR compensatory motions $\Delta\mathbf{R}_c$ of CPD80 proteins, classified according to the compensatory power *P* of the second-site residues. *P* values are partitioned in ten classes q_i, with q_i indicating *P* values comprised between the (*i* -1)-th and *i*-th 10-quantile. A t-test comparison was performed between the first (q_1) and last (q_{10}) RMSIP distributions, which are found different with a level of significance indicated by the orange stars (*** p-value <0.001).

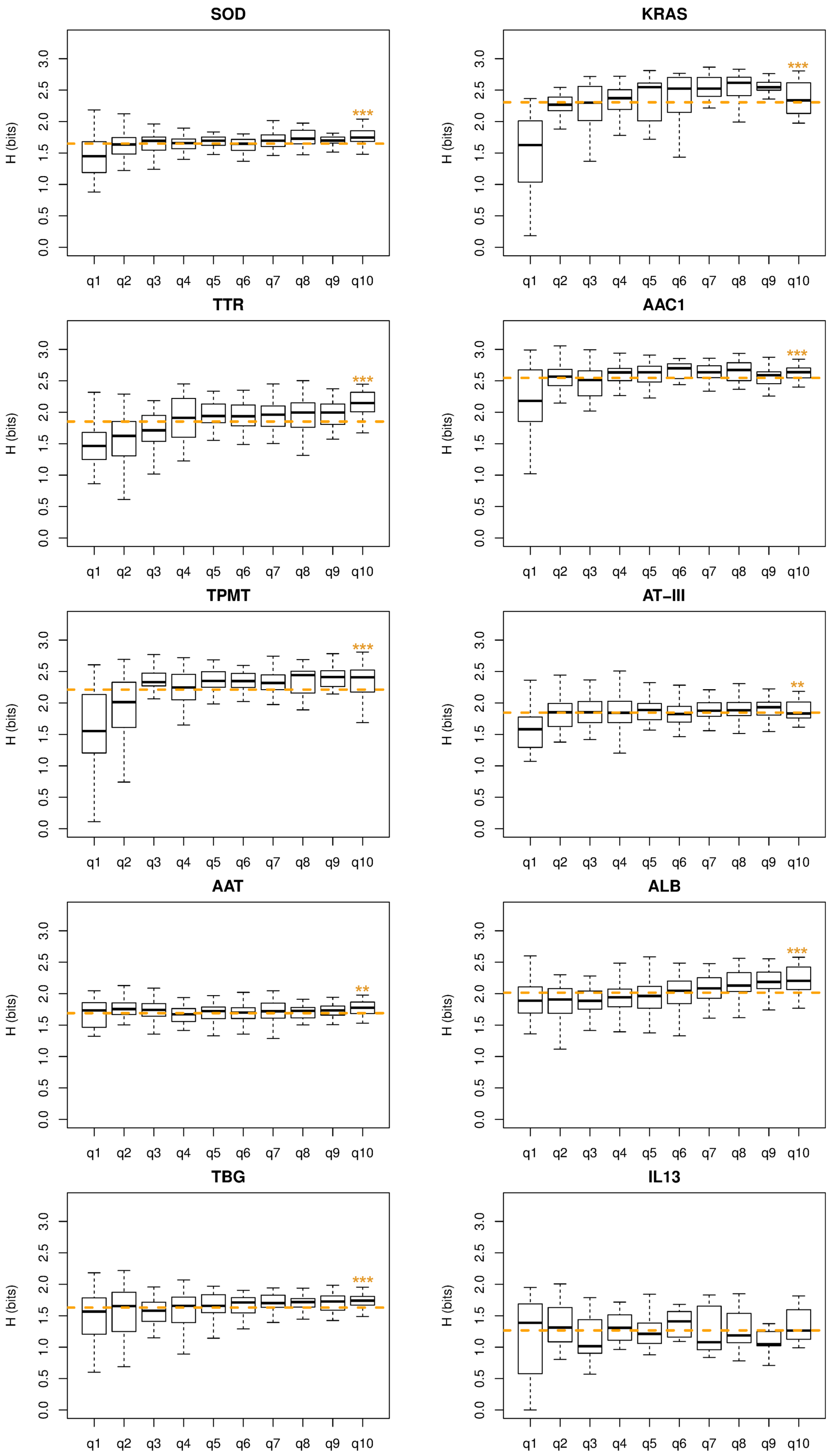

Figure S8. Distributions of dominant normal-mode Shannon Entropy (H) values for the FF compensatory motions Δ**R**c of CPD80 proteins, classified according to the compensatory power *P* of the second-site residues. *P* values are partitioned in ten classes q_i, with q_i indicating *P* values comprised between the (*i* -1)-th and *i*-th 10-quantile. A t-test comparison was performed between the first (q_1) and last (q_{10}) distributions of H values, which are found different with a level of significance indicated by the orange stars (*** p-value <0.001, ** 0.001 ≤ p-value <0.01, * 0.01 ≤ p-value <0.05).

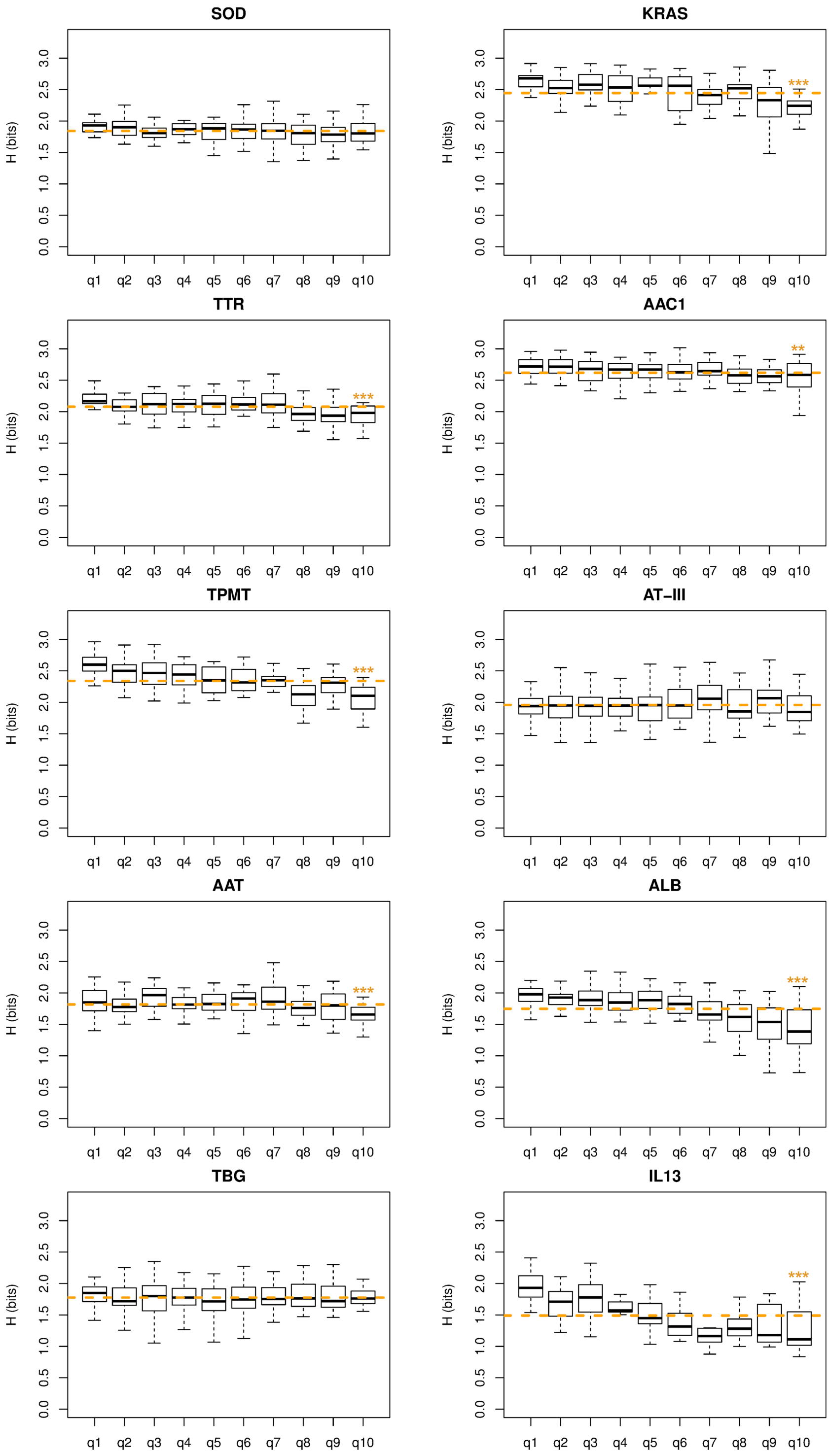

Figure S9. Distributions of dominant normal-mode Shannon Entropy (H) values for the FR compensatory motions $\Delta\mathbf{R}_c$ of CPD80 proteins, classified according to the compensatory power *P* of the second-site residues. *P* values are partitioned in ten classes q_i, with q_i indicating *P* values comprised between the (*i* -1)-th and *i*-th 10-quantile. A t-test comparison was performed between the first (q_1) and last (q_{10}) distributions of H values, which are found different with a level of significance indicated by the orange stars (*** p-value <0.001, ** 0.001 ≤ p-value <0.01, * 0.01 ≤ p-value <0.05).

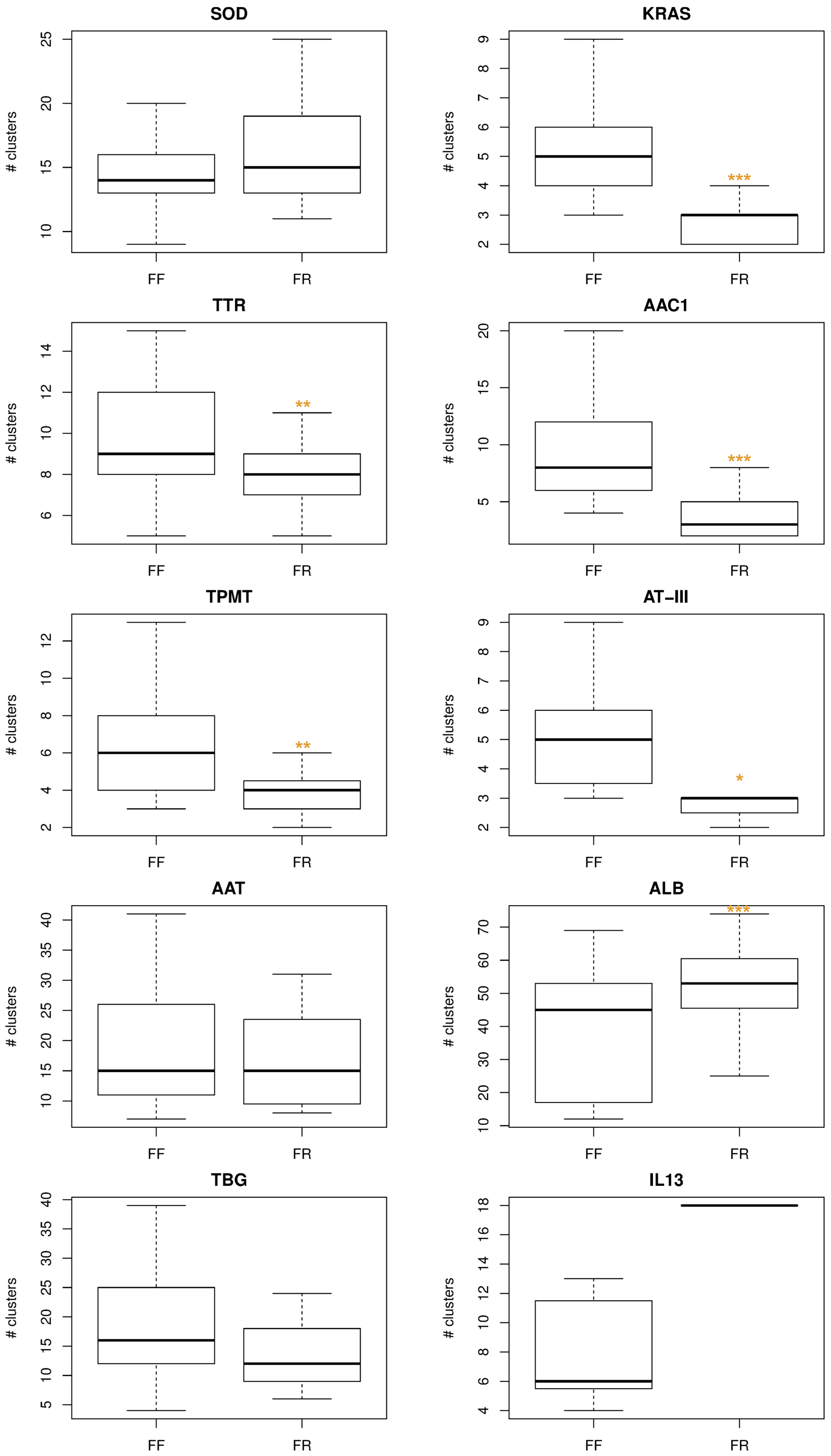

Figure S10. Distribution of the number of clusters of rescued residues for FF and FR rescue sites for CPD80 proteins.

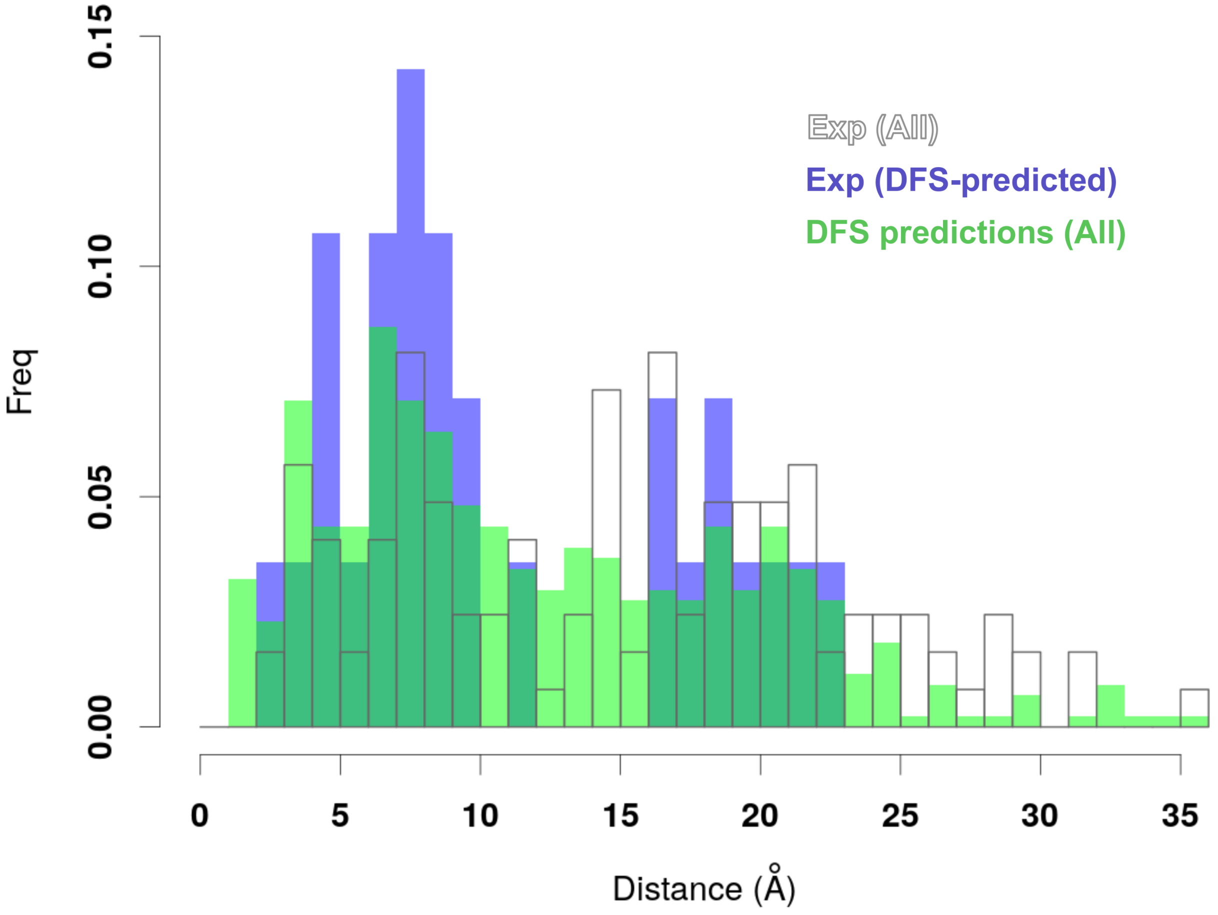

Figure S11. Distribution of p53 pathogenic site(PS)-rescue site(RS) distances for experimental (PS, RS) pairs (white bars). The distributions obtained by considering only the experimental pairs predicted by DFS (blue) and all the pairs predicted by DFS (green) are also reported. Inter-residue distances are calculated as the minimum over all the pairs of non-hydrogen atoms of the two residues. Distributions were normalised so that they have the same area.
